# Ionizing radiation response of primary normal human lens epithelial cells

**DOI:** 10.1371/journal.pone.0181530

**Published:** 2017-07-26

**Authors:** Nobuyuki Hamada

**Affiliations:** Radiation Safety Research Center, Nuclear Technology Research Laboratory, Central Research Institute of Electric Power Industry (CRIEPI), Komae, Tokyo, Japan; Northwestern University Feinberg School of Medicine, UNITED STATES

## Abstract

Whilst the cataractogenic potential of ionizing radiation has been known for over the past 120 years, little is known about radiation responses of lens cells. Our previous work was the first to evaluate the radiosensitivity of lens cells with the clonogenic assay, documenting that the survival of HLEC1 human lens epithelial cells is comparable to that of WI-38 human lung fibroblasts. Moreover, HLEC1 cells were found to contain subsets where irradiation stimulates proliferation or facilitates formation of abortive colonies with fewer cells than human fibroblasts. This study aims to gain insights into these mechanisms. Irradiation of HLEC1 cells with 10% survival dose caused a growth delay but did not reduce viability. HLEC1 cells at high cumulative population doubling level were more susceptible to radiogenic premature senescence than WI-38 cells. Concerning p53 binding protein 1 (53BP1) foci, HLEC1 cells harbored less spontaneous foci but more radiogenic foci than in WI-38 cells, and the focus number returned to spontaneous levels within 48 h postirradiation both in HLEC1 and WI-38. The chemical inhibition of DNA repair kinases ataxia telangiectasia mutated, DNA-dependent protein kinase or both delayed and attenuated the appearance and disappearance of radiogenic 53BP1 foci, increased radiogenic premature senescence and enhanced clonogenic inactivation. The DNA microarray analysis suggested both radiogenic stimulation and inhibition of cell proliferation. Treatment with conditioned medium from irradiated cells did not change growth and the plating efficiency of nonirradiated cells. These results partially explain mechanisms of our previous observations, such that unrepaired or incompletely repaired DNA damage causes a growth delay in a subset of HLEC1 cells without changing viability through induction of premature senescence, thereby leading to clonogenic inactivation, but that growth is stimulated in another subset via as yet unidentified mechanisms, warranting further studies.

## Introduction

Röntgen’s discovery of X-rays in 1896 was followed by observations of ionizing radiation cataracts, of which first case was reported in animals in 1897 and in humans in 1903 [1,2]. In its latest 2007 basic recommendations, the International Commission on Radiological Protection (ICRP) writes that gonads, bone marrow and the crystalline lens of the eye are among the most radiosensitive tissues in the body [3]. ICRP has classified radiation cataracts as tissue reactions (formerly called nonstochastic or deterministic effects) with a dose threshold below which no effect would occur, and has recommended an equivalent dose limit for the ocular lens of workers and public to prevent radiation cataracts [4,5]. Consideration of recent epidemiological evidence led ICRP to recommend in 2011 a threshold of 0.5 Gy (independent of rate of dose delivery and assuming progression of detectable opacities into vision-impairing cataracts) and an occupational equivalent dose limit for the lens of 20 mSv/year, averaged over defined periods of 5 years, with no single year exceeding 50 mSv): these are significant reductions from previously recommendations (i.e., a threshold for detectable opacities of 0.5–2 Gy for acute exposure and 5 Gy for highly fractionated or protracted exposures, a threshold for vision-impairing cataracts of 2–10 Gy for acute exposure and >8 Gy for highly fractionated or protracted exposures, and an occupational equivalent dose limit for the lens of 150 mSv/year) [6,7]. Thus, the lens is now considered much more radiosensitive than previously thought, but its mechanisms remain incompletely understood [8,9].

Lens epithelial cells (LECs) are the only proliferative population among the lenticular structures and have long been regarded as target cells for radiation cataractogenesis [6,10]. Our previous work was the first to evaluate the radiosensitivity of lens cells with the clonogenic assay, and demonstrated that the survival of HLEC1 human LECs and WI-38 human lung fibroblasts following irradiation is similar [11]. Furthermore, HLEC1 cells were found to contain various subsets with differing vulnerability to radiogenic inactivation of clonogenic potential, such that while some cells irradiated at ≥2 Gy form clonogenic colonies with more cells than those arising from sham-irradiated cells, other irradiated cells form abortive colonies with less cells than those arising from irradiated fibroblasts [11]. These findings suggest that irradiation stimulates and inactivates proliferation (at least at ≥2 Gy), but were predicated only on the analysis of colonies formed at 14 days postirradiation. This study therefore aims at gaining insights into mechanisms underpinning these previous observations, and cellular responses occurring at earlier time points were analyzed to this end. This study is the first to report in irradiated lens cells, premature senescence, changes in gene expression profiles, and the impact of inhibition of DNA repair kinases ataxia telangiectasia mutated (ATM) and DNA-dependent protein kinase (DNA-PK) on several endpoints including p53 binding protein 1 (53BP1) foci as a maker of DNA double strand break (DSB) repair, and clonogenic survival.

## Materials and methods

### Cell cultures

HLEC1 primary normal human diploid LECs were passaged in EpiCM, and WI-38 primary normal human diploid lung fibroblasts were subcultured in Dulbecco’s modified Eagle’s medium (DMEM) supplemented with 10% fetal bovine serum (FBS), as described [11]. HLEC1 and WI-38 were purchased from the ScienCell Research Laboratories (Carlsbad, CA) and American Type Culture Collection (CCL-75, Manassas, VA), respectively. 293FT human embryonic kidney cells that stably express the simian virus 40 (SV40) large T antigen and the neomycin resistant gene (Neo^R^) were subcultured in DMEM containing 10% FBS. All cell cultures were maintained at 37°C in a humidified atmosphere of 5% CO_2_ in air, unless otherwise specified.

### Irradiation of HLEC1 and WI-38

Cells were irradiated at room temperature with X-rays at the mean dose rate of 0.42–0.45 Gy/min from an X-ray generator (MBR-1505R2, Hitachi Medico, Tokyo, Japan) operated at 150 kV and 5 mA with a 1-mm aluminum plus 0.2-mm copper filter, as described [11]. For HLEC1 and WI-38, 3.53 Gy and 3.25 Gy were used, respectively, as the dose needed to reduce the surviving fraction to one-tenth (10% survival dose, *D*_10_). These *D*_10_ doses were previously determined by the clonogenic assay, where cells were replated for colony formation within 1 h postirradiation [11]. Control cells were sham-irradiated and handled in parallel with the test cells.

### Cell proliferation and dye exclusion assays in HLEC1

At 24 h prior to irradiation, 1 x 10^5^ cells were plated into a 25 cm^2^ tissue culture flask (T25). At 8 h, 1, 3, 5 or 7 days after *D*_10_ irradiation, culture supernatants were collected, and cells were washed once with Mg^2+^- and Ca^2+^-free phosphate buffered saline (PBS^–^), trypsinized, suspended in culture supernatants, pelleted, resuspended, and counted with a Coulter Z1 particle counter to draw the growth curve. The population doubling (PD) time (*T*_D_) in h was calculated as 24/*q*, when growth curves were fitted against the data points in the exponential growth phase to *y* = s*e^*qx*^ where *y*, *x*, *q* and *s* are cell numbers, time (days), slope and intercept, respectively. Viability was evaluated with a dye exclusion assay, where cell suspensions were mixed with 0.4% trypan blue, and trypan blue-positive cells were counted as nonviable cells with a Countess automated cell counter (C10227, Invitrogen, Carlsbad, CA).

### Senescence-associated β-galactosidase (SA-β-gal) staining in HLEC1, WI-38 and WI-38/hTERT

SA-β-gal is a marker of cellular senescence [12], which was stained using a kit (9890S, Cell Signaling Technology, Danvers, MA) according to the manufacturer’s instruction. To stain prematurely senescent cells, 4 x 10^3^ cells were plated into a 35 mm dish at 24 h prior to irradiation with 1, 2, *D*_10_ or 8 Gy of X-rays. At 7 days postirradiation, cells were washed once with PBS^–^, fixed for 15 min, washed twice with PBS^–^, stained at 37°C, and counted under a Nikon ECRIPSE TS100 microscope. Spontaneous, replicatively senescent cells were stained at 7 or 8 days after plating into a 35 mm dish (2 x 10^4^ cells for HLEC1, 1 x 10^5^ cells for WI-38, or 4 x 10^3^ cells for WI-38/hTERT, see below for details of WI-38/hTERT).

### An attempt to immortalize HLEC1 and WI-38 with the human telomerase catalytic subunit (hTERT)

The pCL vector system [13,14] was used for production of recombinant retroviruses, where HLEC1 and WI-38 served as target cells and 293 FT served as packaging cells. At 24 h after plating (2 x 10^6^ cells/10 cm dish), 293 FT was colipofected with the packaging plasmid pCL-Ampho and the expression vector pCLXSN (for expression of Neo^R^) or pCLXSN-hTERT (for expression of hTERT and Neo^R^), using Lipofectamine 2000 (11688–027, Invitrogen) and Opti-MEM I reduced serum medium (31985–062, Gibco, Grand Island, NY). After colipofection, medium was changed at 24 h, and supernatants were collected at 48–96 h. Supernatants filtered through a 0.45 μm filter were stored in aliquots at –80°C. Retrovirus titer was determined by infecting HLEC1 and WI-38 with tenfold serial dilutions of the supernatant and by selecting resistant colonies with G418 at 400 μg/ml (A1720-5G, Sigma, St Louis, MO). The titer of the retroviral supernatant was in the order of 10^5^ colony forming units/ml. At 24 h after plating into T25 (9 x 10^4^ cells for HLEC1 and 1 x 10^5^ cells for WI-38), the retroviral supernatant and 10 μg/ml polybrene (H9268-5G, Sigma) were added. After addition, medium was changed at 24 h, and selection with 400 μg/ml G418 started at 48 h. After treatment with G418 for 6 days, cells were replated into a 75 cm^2^ tissue culture flask (T75). Cells were routinely subcultured in T75 in the presence of 400 μg/ml G418. Neomycin resistant HLEC1 cells due to pCLXSN and pCLXSN-hTERT were named HLEC1/neo and HLEC1/hTERT, respectively. Likewise, those for WI-38 were named WI-38/neo and WI-38/hTERT.

### Determination of replicative lifespan in HLEC1 and WI-38

To evaluate replicative lifespan, cells were serially subcultured. The PD number (PDN) was calculated as log_2_(*N*_H_/*N*_P_), where *N*_P_ and *N*_H_ are cell numbers plated and those harvested, respectively. The cumulative PDN (CPD) level was calculated as the initial PDN plus the PDN increased by additional passages. The end of the replicative lifespan was defined by failure of the population to increase after a minimum of three weeks in culture with weekly refeedings, as described [11]. *T*_D_ (h) was calculated as 24/*q*, when growth curves were fitted against the data points in the exponential growth phase to *y* = *qx* + *s* where *y*, *x*, *q* and *s* are CPD, time (days), slope and intercept, respectively.

### Preparation of metaphase chromosome spreads in WI-38/hTERT

Exponentially growing cells were treated with 25 ng/ml colcemid (Gibco). Following mild trypsinization and mitotic shakeoff, cells were hypotonized with 75 mM KCl, fixed in 3:1 methanol:acetic acid, dropped onto cleaned glass slides, and stained with 6% Giemsa (Wako, Osaka, Japan). Chromosome numbers in 50 or more metaphases were counted under a Nikon ECRIPSE TS100 microscope.

### Indirect immunofluorescence staining of p53-binding protein 1 (53BP1) in HLEC1 and WI-38

The tumor suppressor 53BP1 is a critical regulator of DSB repair, which is recruited to DSB sites to form subnuclear foci [15–17]. 53BP1 foci were visualized with indirect immunofluorescence as follows. At 24 h prior to irradiation, 4 x 10^4^ cells were seeded onto a 35 mm dish containing a 24 mm x 24 mm glass coverslip (Matsunami Glass, Osaka, Japan). At various time points postirradiation, cells were washed thrice with chilled PBS^–^, placed in cold methanol at –20°C for 20 min, soaked in cold acetone for a few seconds, air dried for >10 min, washed thrice with 0.05% Tween 20 in PBS^–^(TPBS), incubated in 10% bovine serum albumin (BSA) in TPBS at room temperature for 20 min (or overnight at 4°C), and washed once with TPBS. Coverslips were reacted with anti-53BP1 rabbit polyclonal antibody (Ab-1, PC712, Calbiochem, Darmstadt, Germany) diluted 1:500 with 1% BSA in TPBS at room temperature for 1 h (or overnight at 4°C), washed thrice with TPBS, reacted with Alexa Fluor 594 goat anti-rabbit IgG secondary antibody (A11037, Molecular Probes) diluted 1:250 with PBS^–^at room temperature for 1 h in the dark, washed twice with TPBS, washed once with PBS^–^, and mounted in ProLong Gold antifade mountant with 4’,6-diamidino-2-phenylindole (P36931, Molecular Probes). Images were captured using an Olympus BX51 fluorescence microscope equipped with an Olympus DP73 charge-coupled device camera and the Olympus imaging software cellSens. The number of foci/cell/Gy was determined as *q* when the dose response curves were fitted against the data points at 0–1 Gy to *y* = *qx* + *s* where *y*, *x*, *q* and *s* are foci/cell, dose (Gy), slope and intercept, respectively, with correlation coefficient squared (*r*^2^) >0.97.

### Treatment of HLEC1 and WI-38 with inhibitors of DNA repair kinases ataxia telangiectasia mutated (ATM) and DNA-dependent protein kinase (DNA-PK)

KU-55933 (2-morpholin-4-yl-6-thianthren-1-yl-pyran-4-one) is a potent, specific ATM kinase inhibitor (ATMi) with a half maximal inhibitory concentration (IC_50_) of 13 nM [18]. KU-55933 (S1092, Selleck, Houston, TX) was dissolved in dimethylsulfoxide (DMSO, D2650, Sigma) at 20 mM, and cells were treated at 10 μM (i.e., 1:2000). NU7411 (8-dibenzothiophen-4-yl-2-morpholin-4-yl-chromen-4-one) is a potent, specific DNA-PK inhibitor (DNA-PKi) with an IC_50_ of 14 nM [19,20]. NU7411 (3712, Tocris Bioscience, Bristol, UK) was dissolved in DMSO at 2 mM, and cells were treated at 1 μM (i.e., 1:2000). Control cells were mock-treated with 0.1% DMSO as a vehicle and manipulated in parallel with the test cells, such that added to the cultures were 0.5 μl/ml of 20 mM KU-55933 and 0.5 μl/ml of DMSO for KU-55933 treatment, 0.5 μl/ml of 2 mM NU7411 and 0.5 μl/ml of DMSO for NU7411 treatment, 0.5 μl/ml of 20 mM KU-55933 and 0.5 μl/ml of 2 mM NU7411 for cotreatment with KU-55933 and NU7411, and 1 μl/ml of DMSO for mock treatment.

For 53BP1 staining, inhibitors were added at 0.5–1 h prior to irradiation, and present until fixation at 0.1–144 h postirradiation. For a “continuous” treatment for SA-β-gal staining, inhibitors were added at 0.5–1.5 h prior to irradiation, and present until fixation at 7 days postirradiation. For a “16 h” treatment for SA-β-gal staining, inhibitors were added at 0.25–2.5 h prior to irradiation and were removed at 16–17 h postirradiation, followed by fixation at 7 days postirradiation.

To investigate the cytotoxic effect of inhibitors on the clonogenic potential, cells were treated for 16–17 h with inhibitors at 24 h after plating of 4 x 10^4^ cells (HLEC1, but 5 x 10^5^ cells only for cotreatment with KU-55933 and NU7411) or 2 x 10^5^ cells (WI-38), and replated into 10 cm dishes in quadruplicate to quadecuplicate for colony formation. For “16 h” and “continuous” treatment groups, colonies were formed for 13 days in the absence and presence of inhibitors, respectively, at which time cells were fixed in methanol and stained with crystal violet. The relative plating efficiency was calculated as the number of clonogenic colonies formed divided by that of plated, and cytotoxitcity was compared as changes in the percent plating efficiency (i.e., 100 multiplied by the relative plating efficiency).

To examine the impact of inhibitors on radiogenic inactivation of clonogenic potential, 4 x 10^4^ cells (HLEC1, but 5 x 10^5^ cells only for cotreatment with KU-55933 and NU7411) or 2 x 10^5^ cells (WI-38) were plated into T25 at 24 h prior to 2 Gy irradiation. Inhibitors were added at 2–2.5 h prior to irradiation and present until cultures were replated into 10 cm dishes in quadruplicate at 16–17 h postirradiation. For a “16 h” treatment group (HLEC1) and a “continuous” treatment group (WI-38), colonies were formed for 13 days in the absence and presence of inhibitors, respectively, at which time cells were fixed and stained. The surviving fraction at 2 Gy (SF2) was calculated as the number of clonogenic colonies formed that was divided by that of irradiated cells plated and multiplied by the relative plating efficiency in sham-irradiated cells.

### DNA microarray analysis in HLEC1

At 3 days prior to irradiation, 5 x 10^5^ cells were plated into T25. At 3 h after irradiation with sham, 0.5 or 4 Gy, and at 8 h after irradiation with sham or 0.5 Gy, culture supernatants were harvested, and cells were washed once with PBS^–^, trypsinized, suspended in culture supernatants, precipitated, washed twice with PBS^–^, precipitated and stored at –80°C. Total RNA was extracted, and its quality was checked with the Agilent 2100 BioAnalyzer (Agilent, Santa Clara, CA). The Agilent Low Input Quick Amp Labeling Kit, One-Color was used for synthesis of Cy3-labeled cRNA from 0.1 μg of total RNA. The Agilent Gene Expression Hybridization Kit was used to hybridize 0.6 μg of Cy3-labeled cRNA to the Agilent SurePrint G3 Human Gene Expression v2 8x60K Microarrays (three glass slides each formatted with eight high-definition 60K arrays). Microarray slides were scanned with the Agilent G2565CA DNA Microarray Scanner, and scanned data were extracted with the Agilent Feature Extraction software. Gene ontology (GO) was analyzed with AmiGO (http://amigo.geneontology.org/amigo), and pathway analysis was conducted using KEGG (Kyoto Encyclopedia of Genes and Genomes, http://www.kegg.jp/kegg/) and IPA (Ingenuity Pathway Analysis, https://www.qiagenbioinformatics.com/products/ingenuity-pathway-analysis/).

### Treatment of HLEC1 with conditioned medium (CM)

At 24 h prior to irradiation, 3 x 10^5^ cells were plated into T25. At 2 h prior to irradiation, DMSO was added at 1 μl/ml (i.e., 0.1%). At 16–17 h after sham or 2 Gy irradiation, cells were replated into 10 cm dishes in quadruplicate (at 500 cells/dish for sham and 1500 cells/dish for 2 Gy). After incubation for 13 days in the presence of 0.1% DMSO, culture supernatants were harvested, filtered through a 20-μm filter, and immediately used as CM to examine the impacts of the 5 and 9 day treatment on cell proliferation and the 14 day treatment on the clonogenic potential. To assess cell proliferation, the medium was replaced with CM at 48 h after plating of 4 x 10^4^ cells into T25 in triplicate, and cells were treated for 5 days at which time cells were counted with a Coulter Z1 particle counter. Likewise, the medium was replaced with CM at 48 h after plating of 4 x 10^3^ cells into T25 in triplicate, and cells were treated for 9 days followed by counting. To evaluate the clonogenic potential, the medium was replaced with CM at 48 h after plating of 4 x 10^2^ cells into T25 in triplicate, and cells were treated for 14 days at which time colonies were fixed and stained.

### Statistical analysis

Data were calculated as the means and standard deviations (SDs) of three or more repeated experiments except where otherwise stated.

**Fig 1:**
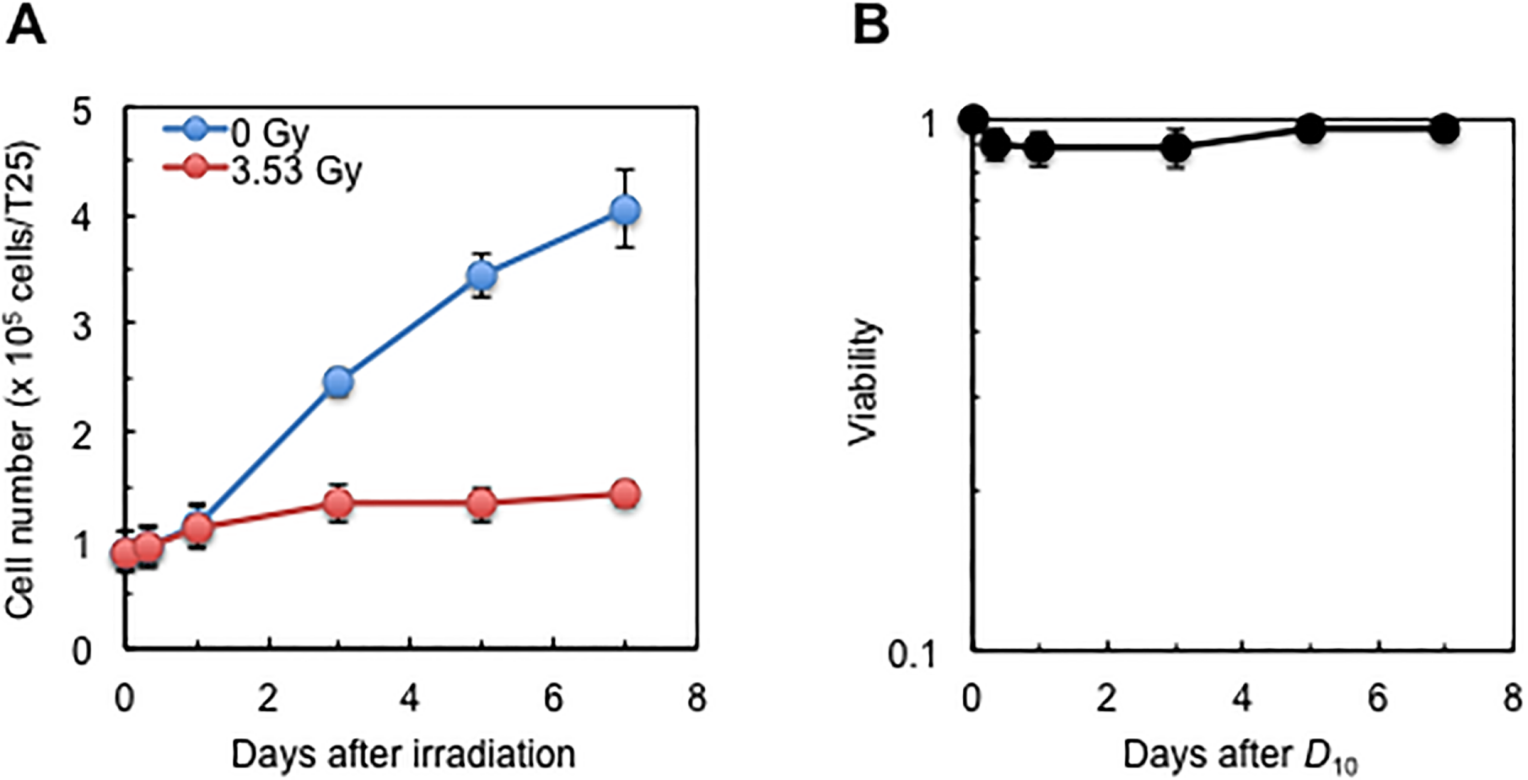
Changes in cell growth and viability of HLEC1 following irradiation with sham or *D*_10_ of X-rays. (A) Growth curve. (B) Viability evaluated with a trypan blue dye exclusion assay. Data are presented as means and SDs of three independent experiments with single measurements, where CPD at the time of irradiation was 14.0 ± 0.3 and dose rate was 0.42 ± 0.01 Gy/min.

**Fig 2:**
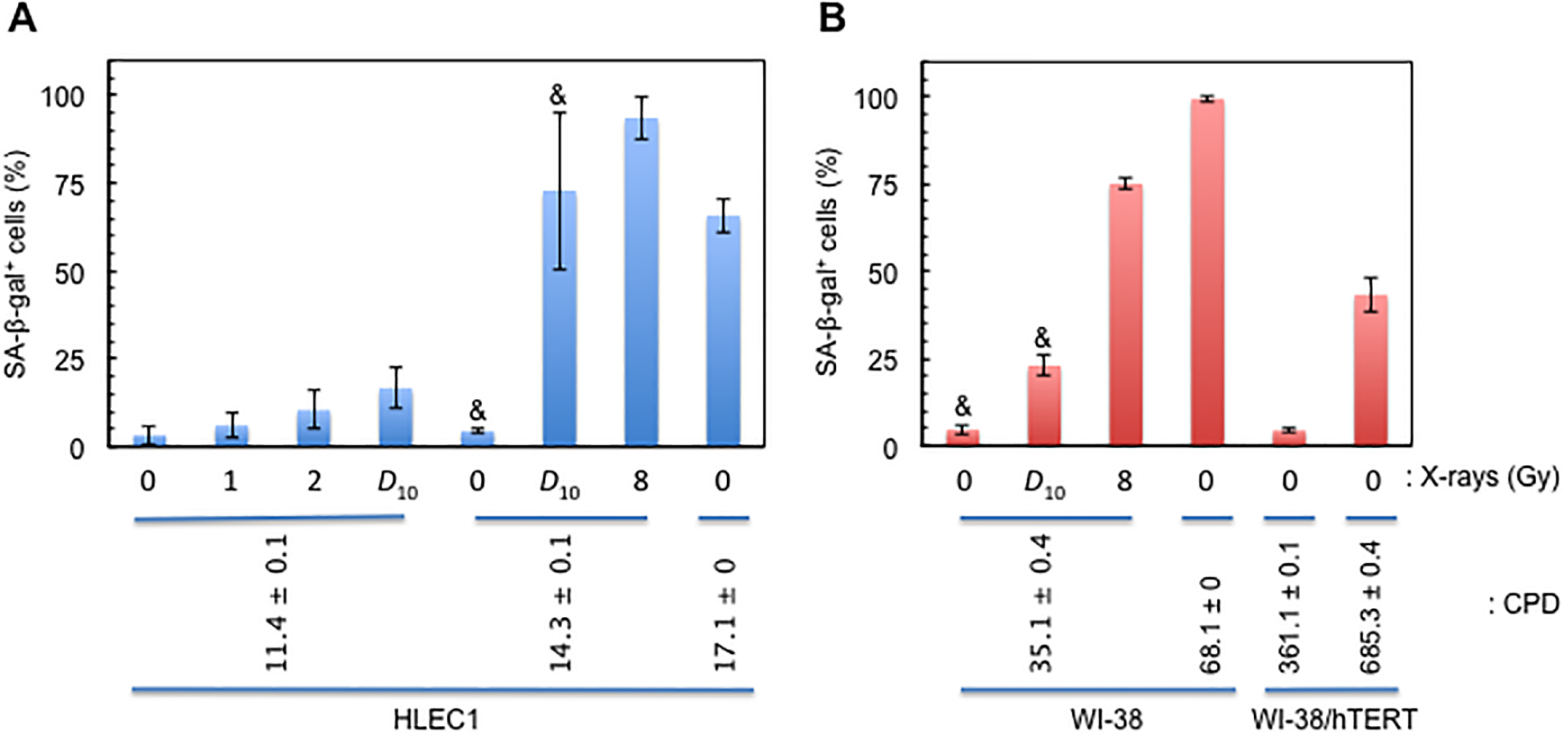
Alterations in SA-β-gal positivity. (A) HLEC1. (B) WI-38 and WI-38/hTERT. CPD shown for HLEC1 at CPD 17.1 ± 0.0 and WI-38/hTERT at CPD 685 ± 0.4 is at the time of plating, and SA-β-gal positivity shown is at 7 days after plating. CPD shown for WI-38 at CPD 68.1 ± 0.0, and WI-38/hTERT at CPD 361 ± 0.1 is at the time of plating, and SA-β-gal positivity shown is at 8 days after plating. CPD shown for HLEC1 at CPD 11.4 ± 0.1 and 14.3 ± 0.1 and WI-38 at CPD 35.1 ± 0.4 is at the time of irradiation with 1, 2, *D*_10_ or 8 Gy (at dose rate of 0.44 ± 0.01, 0.43 ± 0.00 and 0.43 ± 0.01 Gy/min, respectively), and SA-β-gal positivity shown is at 7 days postirradiation. Data represent means and SDs of three independent experiments, where 192–2048 cells were counted for each sample. *These data were taken from [58].

**Fig 3:**
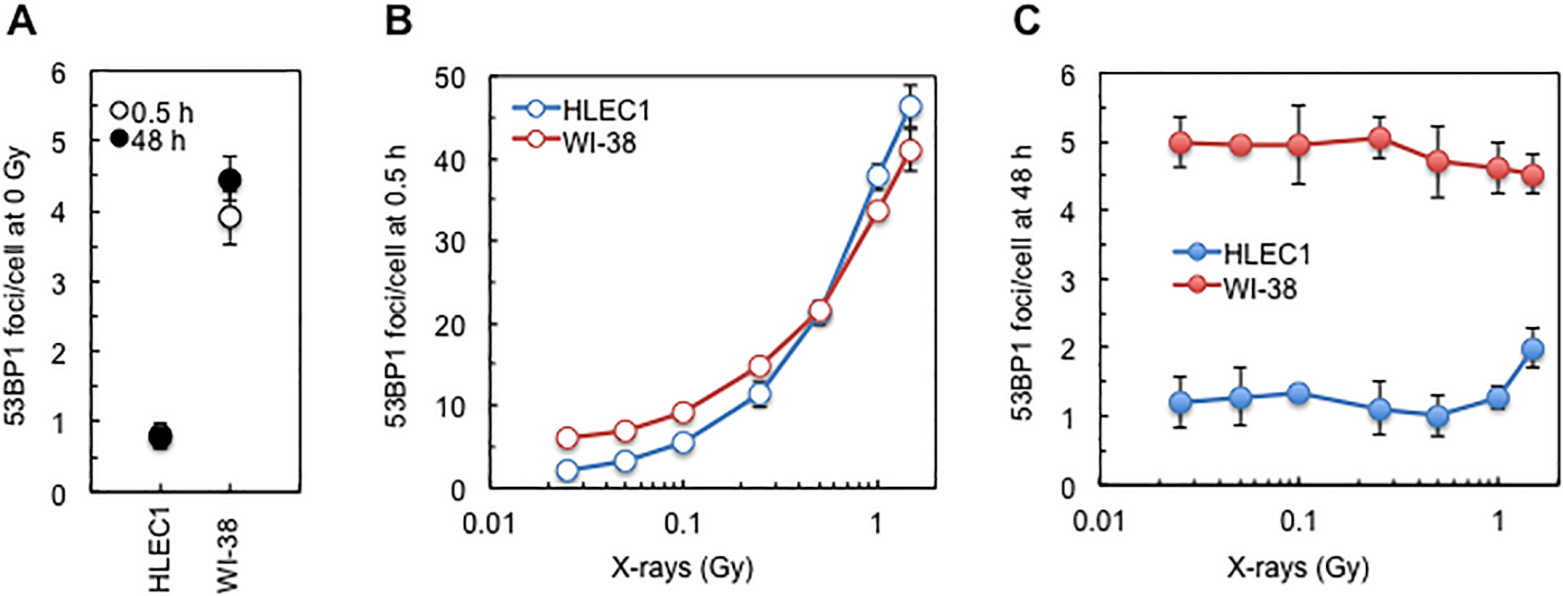
Dose response for 53BP1 focus formation at 0.5 or 48 h after irradiation with 0.025, 0.05, 0.1, 0.5, 1 and 1.5 Gy of X-rays. (A) At 0.5 and 48 h after sham irradiation. (B) At 0.5 h after 0.025–1.5 Gy. (C) At 48 h after 0.025–1.5 Gy. For HLEC1 and WI-38, CPD at the time of irradiation was 14.3 ± 0.1 and 40.0 ± 0.3, and dose rate was 0.42 ± 0.02 Gy/min and 0.44 ± 0.01 Gy/min, respectively. Data shown are means and SDs of three independent experiments, where 100–325 cells were counted for each sample.

**Fig 4:**
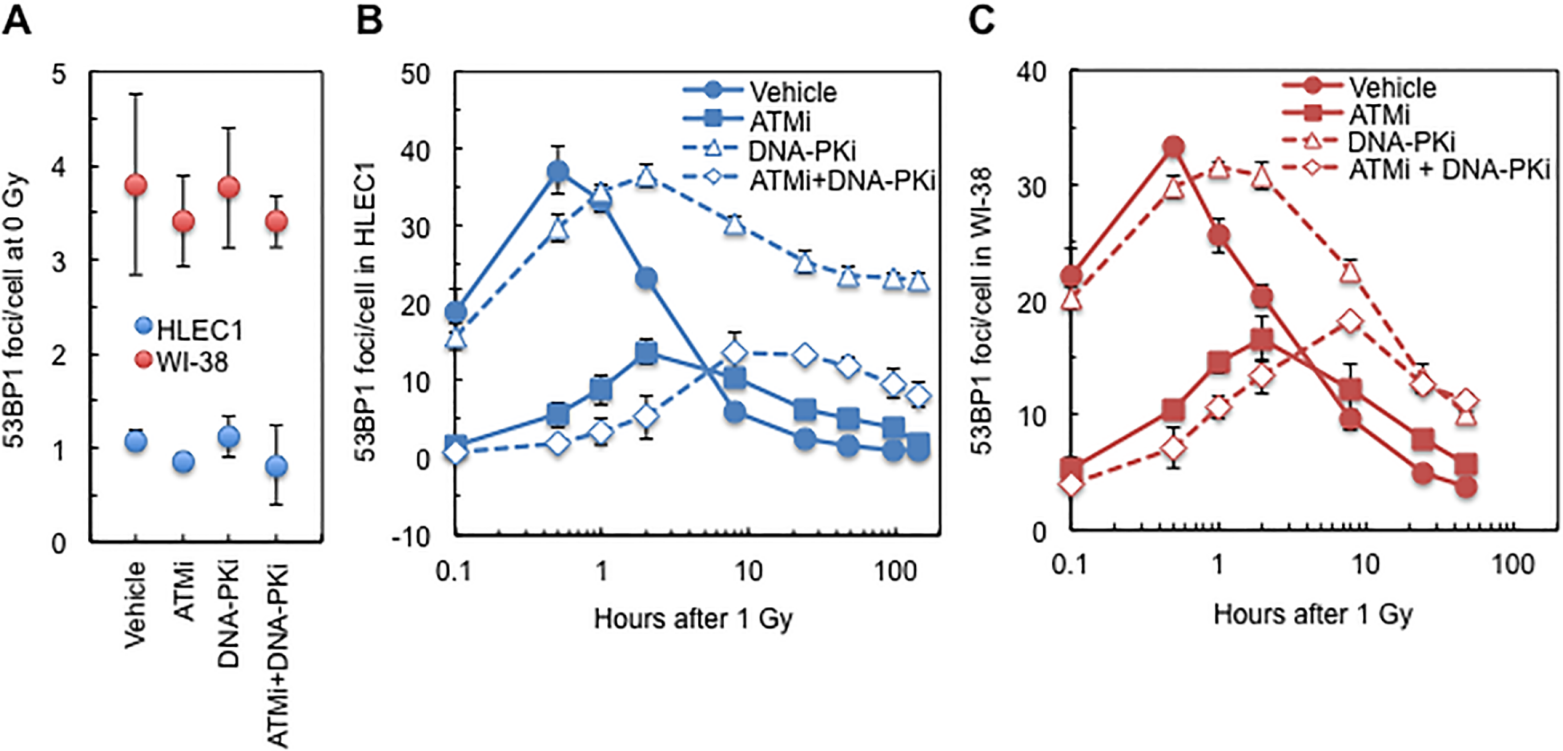
Effect of a continuous treatment with ATMi and/or DNA-PKi on temporal kinetics of 53BP1 focus formation at 0.1, 0.5, 1, 2, 8, 24, 48, 96 or 144 h after 1 Gy irradiation. (A) HLEC1 and WI-38 after sham irradiation. (B) HLEC1 after 1 Gy irradiation. (C) WI-38 after 1 Gy irradiation. For HLEC1 and WI-38, CPD at the time of irradiation was 14.1 ± 0.3 and 39.9 ± 0.4, and dose rate was 0.44 ± 0.00 and 0.43 ± 0.00 Gy/min, respectively. Inhibitors were added at 0.5–1 h prior to irradiation and continued to exist until cells were fixed at indicated times postirradiation. Data represent means and SDs of three independent experiments, where 100–318 cells were counted for each sample.

**Fig 5:**
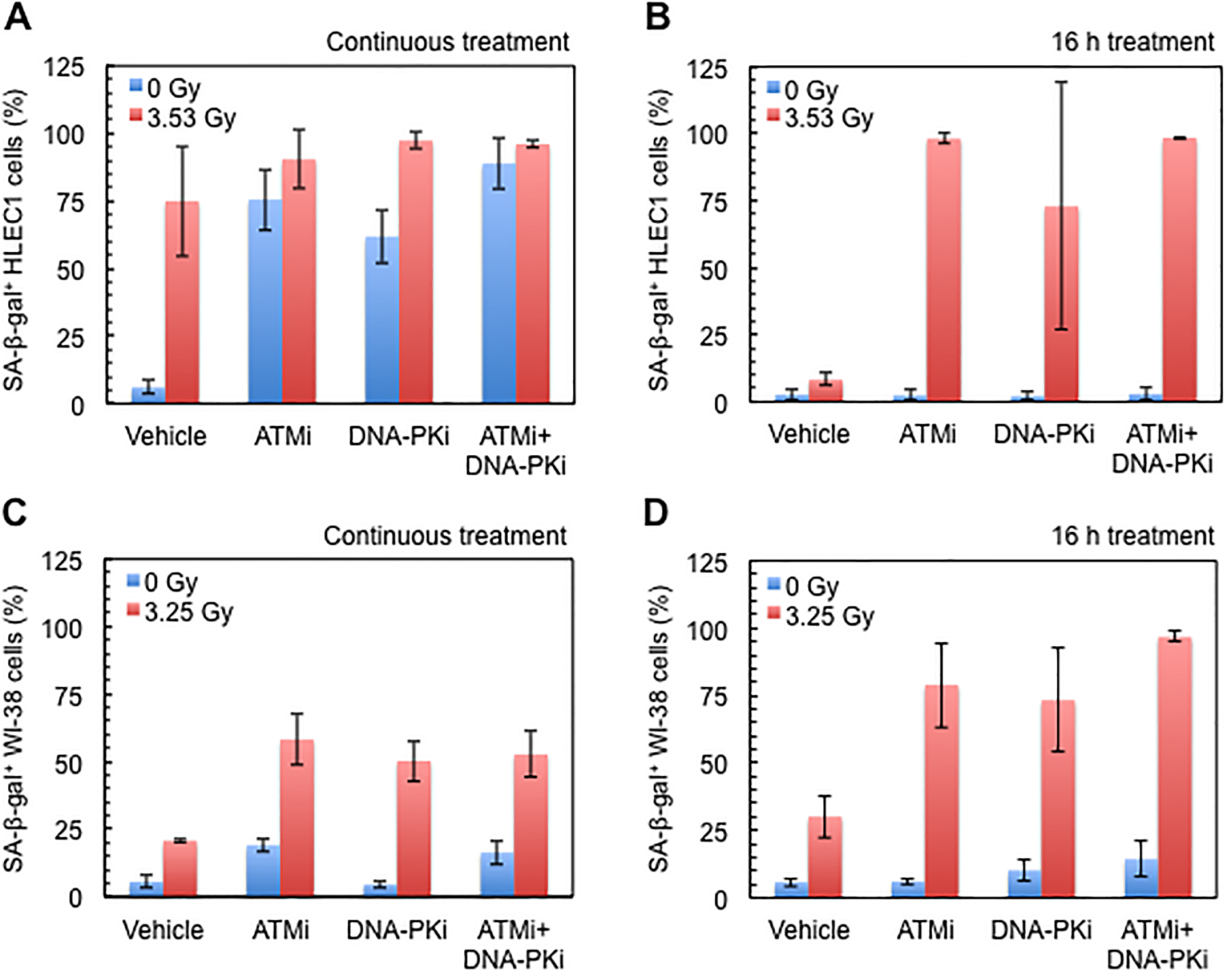
Effect of a continuous or a 16 h treatment with ATMi and/or DNA-PKi on SA-β-gal positivity at 7 days after *D*_10_ irradiation. (A) A continuous treatment of HLEC1. CPD at the time of irradiation was 14.3 ± 0.1, and dose rate was 0.43 ± 0.00 Gy/min. (B) A 16 h treatment of HLEC1. CPD at the time of irradiation was 11.4 ± 0.1, and dose rate was 0.44 ± 0.01 Gy/min. (C) A continuous treatment of WI-38. CPD at the time of irradiation was 35.1 ± 0.2, and dose rate was 0.43 ± 0.00 Gy/min. (D) A 16 h treatment of WI-38. CPD at the time of irradiation was 34.2 ± 0.2, and dose rate was 0.44 ± 0.00 Gy/min. For a continuous treatment, inhibitors were present from 0.5–1.5 h prior to irradiation until fixation at 7 days postirradiation. For a 16 h treatment, inhibitors were present from 0.25–2.5 h prior to irradiation until 16–17 h after irradiation, and were absent until fixation at 7 days postirradiation. Data are presented as means and SDs of three independent experiments, where 100–1298 cells were counted for each sample.

**Fig 6:**
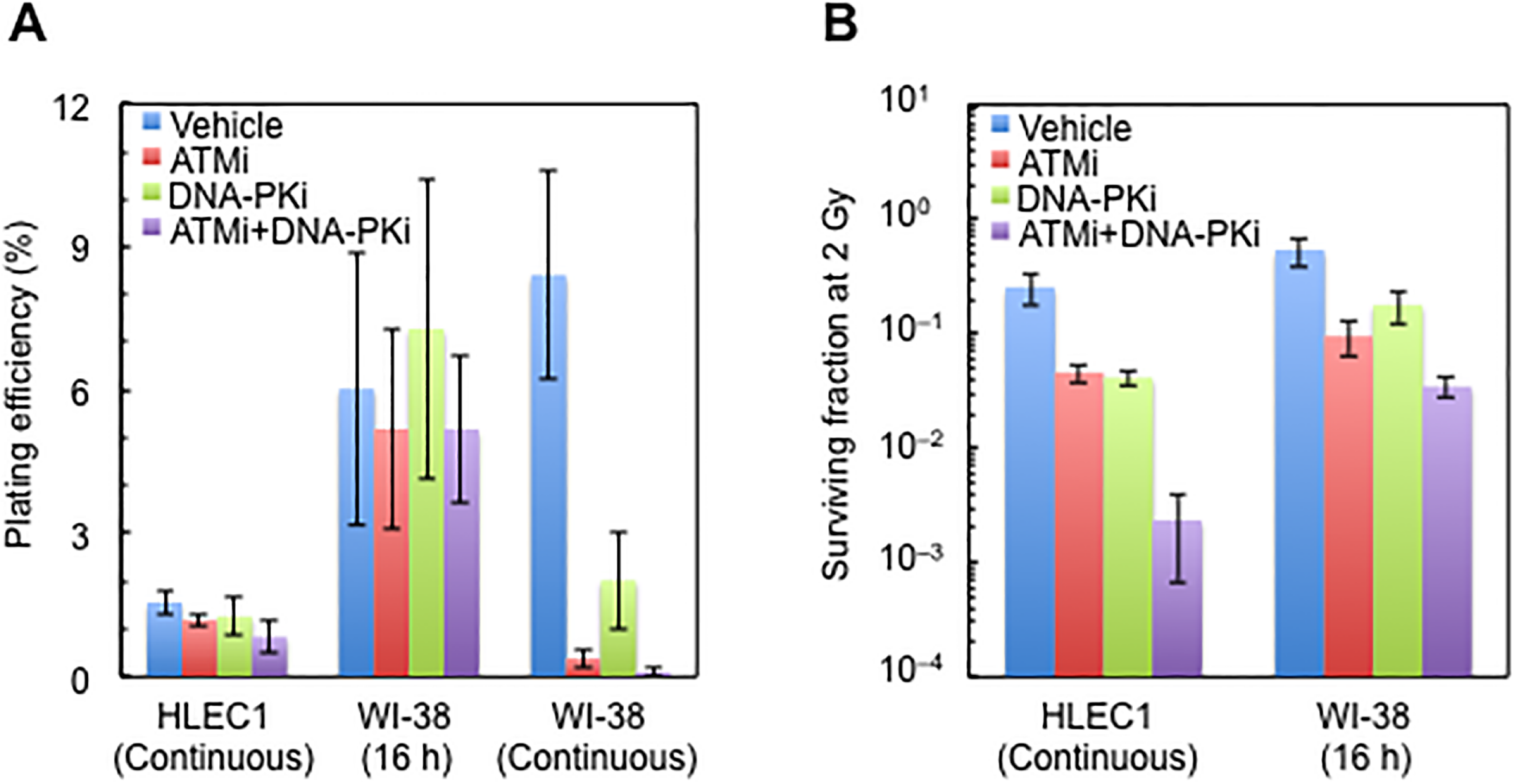
Effect of a continuous or a 16 h treatment with ATMi and/or DNA-PKi on the plating efficiency and survival after 2 Gy irradiation. (A) Plating efficiency. (B) SF2. Cells were replated for colony formation at 16–17 h after 2 Gy, and colonies were formed for 13 days. For a continuous treatment, inhibitors were present from 1–2.5 h prior to irradiation until fixation of colonies. For a 16 h treatment, inhibitors were present from 0.25–2.5 h prior to irradiation until 16–17 h postirradiation, but were absent during colony formation. For a continuous treatment of HLEC1, CPD at the time of plating was 11.2 ± 0.0, and dose rate was 0.45 ± 0.00 Gy/min. For a continuous treatment of WI-38, CPD at the time of plating was 38.7 ± 0.3. For a 16 h treatment of WI-38, CPD at the time of plating was 35.4 ± 0.2, and dose rate was 0.44 ± 0.00 Gy/min. Data shown are means and SDs of three to ten independent experiments with quadruplicate to quadecuplicate measurements.

For the data presented in Figs 1–6, the general linear model analysis of variance (ANOVA) with post hoc Turkey's multiple comparison test was used to determine *p* values, where *p* <0.05 was considered to be significant. In all cases, the suggested models used direct relationships between the result and each of the experimental variables: e.g., result ~ (days, assay, dose) for Fig 1A. Where significant responses were observed, regression with F-test for significance of relationships was also applied. Where appropriate, Student’s *t*-test was also used to determine *p* values, as specified in the Results section.

For the microarray data presented in S1–S11 Tables, *p* values and a false discovery rate (FDR) were determined with Student’s *t*-test, and the Benjamini-Hochberg procedure [21], respectively.

## Results

### *D*_10_ irradiation causes a growth delay without changing viability in HLEC1

Our previous findings showing radiogenic stimulation and inactivation of proliferation in HLEC1 were obtained only from the analysis of colonies formed at 14 days postirradiation [11]. To examine growth changes occurring at earlier time points, HLEC1 was assessed for growth and viability up to 7 days postirradiation. For growth, ANOVA reveals a significant effect of both time and dose, with days 5 and 7 significantly different from the earlier days with *p* all <0.05 (Fig 1A). Furthermore, regression revealed a significant linear relationship between days and dose at 3.53 Gy, with an F-test *p* value of <0.001. At 1–5 days postirradiation, sham- and *D*_10_-irradiated cells had *T*_D_ of 60.0 h and 382 h, respectively (Fig 1A). Viability evaluated with a trypan blue dye exclusion assay did not significantly change throughout the observation period (Fig 1B). These results demonstrate that *D*_10_ irradiation (with which 90% of clonogenic cells are supposed to undergo clonogenic inactivation) leads to a growth delay but does not reduce viability. This suggests a predominant role of a long term cell cycle arrest in contrast to little if any role of apoptosis or other cell death modes that increase membrane permeability. As its possible mechanism, cellular senescence was next assessed.

### HLEC1 at higher CPD is more susceptible to radiogenic premature senescence

Cellular senescence was evaluated with SA-β-gal staining at 7 days postirradiation. Fig 2 shows that irradiation with *D*_10_ and 8 Gy significantly increases SA-β-gal positivity in HLEC1 and WI-38 (ANOVA *p* for dose <0.001 in both cases), and the pairwise testing revealed that SA-β-gal positivity at *D*_10_ and 8 Gy was significantly different from that at 0, 1 and 2 Gy. In HLEC1 (Fig 2A), a difference in SA-β-gal positivity at CPD 11.4 ± 0.1 and 14.3 ± 0.1 was statistically insignificant at 0 Gy (*t*-test *p* = 0.51 between 3.3 ± 2.7% and 4.5 ± 0.9%), but SA-β-gal positivity at CPD 14.3 ± 0.1 (72.9 ± 22.5%) was significantly higher than that at CPD 11.4 ± 0.1 (16.8 ± 6.1%) at *D*_10_ (*t*-test *p* = 0.02). These results suggest that irradiation induces premature senescence in HLEC1 and WI-38, and indicate that HLEC1 at higher CPD is more vulnerable to such radiogenic premature senescence.

### Most cells whose CPD has just reached a peak are SA-β-gal positive in WI-38 but not in HLEC1

To compare the degree of radiogenic premature senescence with that of spontaneous, replicative senescence, SA-β-gal positivity in old cells was examined. In this regard, we previously reported that HLEC1 has *T*_D_ of 66.6 h, and once reaching 17.1, CPD does not increase further at least up to 24 days with three weekly refeedings (S1A Fig), and that WI-38 has *T*_D_ of 32.4 h, and once reaching 69.1, CPD does not increase further at least up to 88 days with twelve weekly refeedings (S1C Fig) [11]. Expectedly, SA-β-gal positivity in WI-38 was 99.4 ± 0.8% at 8 days after CPD reached 68.1 ± 0.0 (Fig 2B). Unexpectedly, however, SA-β-gal positivity in HLEC1 was 65.9 ± 4.8% at 7 days after CPD reached 17.1 ± 0.0 (i.e., a maximum) (Fig 2A), but was increased to 99.5% at 276 days after CPD reached a maximum during which time cells underwent two passages and 36 weekly refeedings (observation from a single experiment where 219 SA-β-gal positive cells were counted). These results show that almost all cells whose CPD has just reached a maximum are SA-β-gal positive in WI-38 but not in HLEC1, and indicate that replicatively senescent HLEC1 takes more time to become SA-β-gal positive than replicatively senescent WI-38.

### Retrovirus-mediated hTERT transduction extends replicative lifespan in WI-38 but not in HLEC1

Not only because replicative lifespan of HLEC1 was short, but also because if its replicative lifespan can be extended, it will be very useful to conduct further analyses (e.g., by cloning clonogenically stimulated cells following irradiation), an attempt was made to prolong replicative lifespan by retrovirus-mediated hTERT transduction. Replicative lifespan was significantly extended in WI-38 (S1D Fig), but not in HLEC1 (S1B Fig). Whilst WI-38/neo reached the end of replicative lifespan at CPD 51.6, WI-38/hTERT continued to divide at least up to CPD 707. WI-38/hTERT had *T*_D_ of 37.6 h at CPD 31.0–707 (*r*^2^ = 0.98995), with possible triphasic changes in growth rate bordering at around CPD 200 and 400, e.g., the first, slowly growing phase with *T*_D_ of 40.6 h at CPD 31.0–200 (*r*^2^ = 0.99498), the second, intermediately growing phase with *T*_D_ of 37.3 h at CPD 200–403 (*r*^2^ = 0.99614), and the third, rapidly growing phase with *T*_D_ of 30.4 h at CPD 403–707 (*r*^2^ = 0.99995). The karyotype analysis of metaphase chromosome spreads confirmed that WI-38/hTERT is diploid both at CPD 367 (in the second phase) and 674 (in the third phase). Post hoc testing showed that there were significant differences (ANOVA *p* <0.001) between all CPD values [e.g., CPD 361 ± 0.1 (in the second phase) and 685 ± 0.4 (in the third phase) in WI-38/hTERT, and CPD 35.1 ± 0.4 and 68.1 ± 0.0 in WI-38], apart from SA-β-gal positivity at CPD 35.1 ± 0.4 in WI-38 vs at CPD 361.1 ± 0.1 in WI-38/hTERT.

Next, to investigate the potential mechanism of premature senescence, the dose response was examined for appearance and disappearance of 53BP1 foci.

### HLEC1 bears less spontaneous and more radiogenic 53BP1 foci than WI-38

In HLEC1 (Fig 3), dose, time and cell type were all significant results with ANOVA *p* <0.001, and there is evidence of an interactive effect between dose, cell and time point (ANOVA *p* <0.001). The number of 53BP1 foci/cell (referred hereinafter to as the focus number) at 0.5 h after 0.1–1.5 Gy was significantly higher than that at 0.5 h after 0 Gy (*p* <0.001), but there were no significant differences between the data points at 48 h. In WI-38 (Fig 3), the focus number at 0.5 h after 0.05–1.5 Gy was significantly higher than that at 0.5 h after 0 Gy (ANOVA *p* ≤0.018), but the focus number at 48 h after 0.025–1.5 Gy was insignificantly different from that at 48 h after 0 Gy (ANOVA *p* all >0.999). At 0 Gy (Fig 3A), the focus number in HLEC1 (0.8 ± 0.0 for 0.5 h and 0.8 ± 0.2 for 48 h) was significantly lower than that in WI-38 (3.9 ± 0.4 for 0.5 h and 4.5 ± 0.3 for 48 h) (ANOVA *p* = 0.024 for 0.5 h and ANOVA *p* = 0.002 for 48 h). At 0.5 h postirradiation (Fig 3B), the focus number at 1 Gy and foci/cell/Gy (the slope of the linearly fitted dose response curve at 0–1 Gy with 0.993< *r*^2^ <0.999 for HLEC1 and 0.979< *r*^2^ <0.988 for WI-38) was significantly higher in HLEC1 than in WI-38 (*t*-test *p* = 0.02 between 37.8 ± 1.6 and 33.6 ± 1.0 foci/cell at 1 Gy, and *t*-test *p* = 0.001 between 37.0 ± 1.5 and 28.9 ± 0.8 foci/cell/Gy). These results show that: (*i*) HLEC1 possesses less spontaneous foci than WI-38; (*ii*) irradiation produces more foci in HLEC1 than in WI-38; and (*iii*) radiogenic foci disappear within 48 h both in HLEC1 and WI-38.

Next, to investigate the role of ATM and DNA-PK, the impact of ATMi and DNA-PKi on 53BP1 focus formation, SA-β-gal positivity and the survival was examined, where DMSO was used for mock treatment as a vehicle.

### Appearance and disappearance of radiogenic 53BP1 foci in HLEC1 and WI-38 are delayed by DNA-PKi, and are delayed and attenuated by ATMi, which are further exacerbated by combination of ATMi and DNA-PKi

Fig 4A and 4B show the temporal kinetics of 53BP1 foci in HLEC1 subjected to sham or 1 Gy irradiation and a continuous inhibitor treatment. In sham-irradiated cells (Fig 4A), there were no significant differences between the four treatment groups (i.e., vehicle, ATMi, DNA-PKi and ATMi+DNA-PKi). In mock-treated cells (Fig 4B), time and treatment were both significant factors (ANOVA *p*< 0.001), and there was also an indication of an interaction effect between these factors (ANOVA *p* = 0.001). The focus number in irradiated cells peaked at 0.5 h postirradiation (37.2 ± 3.1 foci/cell), and was significantly higher at 0.1–8 h postirradiation (ANOVA *p* ≤0.013) but not at 24–144 h (ANOVA *p* ≥0.999) than sham-irradiated cells. In ATMi-treated cells, the focus number in irradiated cells peaked at 2 h postirradiation (13.7 ± 1.6 foci/cell), and was significantly higher at 0.5–24 h postirradiation (ANOVA *p* ≤0.025) but not at 0.1 h nor at 48–144 h (ANOVA *p* >0.072) than sham-irradiated cells. In DNA-PKi-treated cells, the focus number in irradiated cells peaked at 2 h postirradiation (36.5 ± 1.5 foci/cell), and was significantly higher at 0.1–144 h postirradiation (ANOVA *p* <0.001) than sham-irradiated cells. In ATMi+DNA-PKi-treated cells, the focus number in irradiated cells peaked at 8 h postirradiation (13.6 ± 2.7 foci/cell), and was significantly higher at 8–144 h postirradiation (ANOVA *p* <0.001) but not at 0.1–2 h (*p* ≥0.051) than sham-irradiated cells. The peak focus number in DNA-PKi-treated, irradiated cells was insignificantly different from that in mock-treated, irradiated cells (*t*-test *p* = 0.76). The peak focus number in ATMi- and ATMi+DNA-PKi-treated cells was significantly lower than that in mock-treated cells (*t*-test *p* = 0.0003 and 0.01, respectively). The peak focus number in ATMi-treated cells was insignificantly different from that in ATMi+DNA-PKi-treated cells (*t*-test *p* = 0.94). These results show that the appearance and disappearance of radiogenic 53BP1 foci are delayed by DNA-PKi, and are delayed and attenuated by ATMi, which are further exacerbated by combination of ATMi and DNA-PKi, and indicate the involvement of ATM and DNA-PK in DSB repair in HLEC1. The response of WI-38 (Fig 4A and 4C) was similar to that of HLEC1 (Fig 4A and 4B), although there was a significant difference between the HLEC1 and WI-38 responses in sham-irradiated cells (ANOVA *p* <0.001, Fig 4A).

### ATMi, DNA-PKi or both similarly increase spontaneous and radiogenic SA-β-gal positivity in HLEC1 and WI-38, which are more manifested at higher CPD in HLEC1

Fig 5A shows SA-β-gal positivity in HLEC1 subjected to a continuous inhibitor treatment and *D*_10_ irradiation at CPD 14.3 ± 0.1. ANOVA reveals that there were significant effects of dose and treatment condition (ANOVA *p* both <0.001), with post hoc testing revealing a significant difference only between the vehicle and the other treatments, and there was also evidence of an interaction effect between dose and treatment condition (ANOVA *p* <0.001): in sham-irradiated cells, SA-β-gal positivity in ATMi-, DNA-PKi- and ATMi+DNA-PKi-treated cells was significantly higher than that in mock-treated cells (*t*-test *p* <0.001). In irradiated cells, SA-β-gal positivity in ATMi-, DNA-PKi- and ATMi+DNA-PKi-treated cells was insignificantly different from that in mock-treated cells (ANOVA *p* >0.27). A difference between irradiated and sham-irradiated cells was significant in mock- and DNA-PKi-treated cells (ANOVA *p* ≤0.013), but not in ATMi- and ATMi+DNA-PKi-treated cells (ANOVA *p* >0.657).

Fig 5B shows SA-β-gal positivity in HLEC1 subjected to a 16 h inhibitor treatment and *D*_10_ irradiation at CPD 11.4 ± 0.1. ANOVA reveals that there were significant effects of dose and treatment condition (ANOVA *p* both ≤0.001), with post hoc testing revealing a significant difference only between the vehicle and the other treatments, and there was also evidence of an interaction effect between dose and treatment condition (ANOVA *p* = 0.001): in sham-irradiated cells, SA-β-gal positivity in ATMi-, DNA-PKi- and ATMi+DNA-PKi-treated cells was insignificantly different from that in mock-treated cells (ANOVA *p* >0.99). In irradiated cells, SA-β-gal positivity in ATMi-, DNA-PKi- and ATMi+DNA-PKi-treated cells was significantly higher than that in mock-treated cells (ANOVA *p* ≤0.003). A difference between irradiated and sham-irradiated cells was significant in ATMi-, DNA-PKi- and ATMi+DNA-PKi-treated cells (ANOVA *p* ≤0.01), but not in mock-treated cells (ANOVA *p* = 0.999).

For comparison between Fig 5A and 5B, HLEC1 used for a continuous inhibitor treatment had a significantly higher CPD than those used for a 16 h treatment (CPD 14.3 ± 0.1 vs 11.4 ± 0.1, *t*-test *p* = 0.00001). SA-β-gal positivity in mock-treated, sham-irradiated cells subjected to a continuous treatment was insignificantly different from that to a 16 h treatment (6.0 ± 2.4% vs 2.7 ± 2.0%, ANOVA *p* >0.999), but SA-β-gal positivity in mock-treated, irradiated cells subjected to a continuous treatment was significantly higher than that to a 16 h treatment (74.9 ± 20.4% vs 8.2 ± 2.4%, ANOVA *p* = 0.001). This reconfirms that HLEC1 at higher CPD is more susceptible to radiogenic premature senescence (as demonstrated in Fig 2A). SA-β-gal positivity in ATMi-, DNA-PKi- or ATMi+DNA-PKi-treated, sham-irradiated cells was significantly higher than that in mock-treated, sham-irradiated cells for a continuous treatment, but not for a 16 h treatment (ANOVA *p* = 0.005 for a 16 h treatment vs a continuous treatment), suggesting that HLEC1 at higher CPD is more susceptible to senescence induced by inhibitors. Unlike the case for 53BP1 focus formation (Fig 4B), there was no difference in SA-β-gal positivity among ATMi-, DNA-PK-i and ATMi+DNA-PKi-treated cells. These results indicate the involvement of ATM and DNA-PK in spontaneous and radiogenic SA-β-gal positivity. Although ANOVA reveals a significant effect of cell type (ANOVA *p* <0.001), the response of WI-38 (Fig 5C) was similar to that of HLEC1 (Fig 5A), and there was little difference between continuous and 16 h treatments of WI-38 irradiated at similar CPD (Fig 5C and 5D). Overall, ANOVA reveals a significant effect of each of the experimental factors.

### ATMi and DNA-PKi have similar radiosensitizing effects to HLEC1 and WI-38, which are augmented by combination of ATMi and DNA-PKi

For HLEC1, there was no significant effect on plating efficiency of continuous treatment compared to the vehicle (Fig 6A). SF2 of ATMi-, DNA-PKi- and ATMi+DNA-PKi-treated cells was significantly lower than that of mock-treated cells (ANOVA *p* all <0.001). The differences in SF2 between ATMi-, DNA-PKi- and ATMi+DNA-PKi-treated cells were insignificant (ANOVA *p* all >0.165). These results show that ATMi and DNA-PKi have similar radiosensitizing effects to HLEC1, which are further enhanced by combination of ATMi and DNA-PKi.

For WI-38, a 16 h treatment was used because the plating efficiency was significantly low in WI-38 subjected to a continuous ATMi+DNA-PKi treatment (Fig 6A), and a trend in the survival of WI-38 following a 16 h treatment was similar to that of HLEC1 following a continuous treatment (Fig 6B), with significant differences again observed between each of the three treatment conditions (i.e., ATMi-, DNA-PKi- and ATMi+DNA-PKi) and the mock treatment (ANOVA *p* all <0.002). To look for other mechanisms, gene expression profiles were next analyzed.

### Gene expression profiles in HLEC1 do not change at 3 or 8 h after 0.5 Gy, but those at 3 h after 4 Gy suggest changes in genes related to cell proliferation

The RNAs extracted from HLEC1 at 3 h after irradiation with sham, 0.5 or 4 Gy, and at 8 h after irradiation with sham or 0.5 Gy were subjected to the DNA microarray analysis. Unexpectedly, irradiation did not dramatically change gene expression profiles, as is evident from the results of hierarchical clustering (S2 Fig).

S1 Table demonstrates that unlike thousands of probes with significant changes at *p* <0.05 (and FDR ≤1), no probes underwent significant changes at *p* <0.05 and FDR < 0.1 except at 3 h after 4 Gy vs 0 Gy. At *p* <0.05 and FDR <0.05 at 3 h after 4 Gy vs 0 Gy, 10 genes changed (S1 Table), of which 4 and 2 genes were up- and downregulated <1.5 fold, respectively, and 2 and 2 genes were up- and downregulated >1.5 fold, respectively (these 10 genes listed in S2 Table). At *p* <0.05 and FDR <0.1 at 3 h after 4 Gy vs 0 Gy, 356 genes changed (S1 Table), among which 128 and 193 genes were up- and downregulated <1.5 fold, respectively (these 321 genes listed in S4 Table), and 22 and 13 genes were up- and downregulated >1.5 fold, respectively (these 35 genes listed in S3 Table).

Many of the genes whose expression changed at *p* <0.05 and FDR <0.1 at 3 h after 4 Gy vs 0 Gy were related to cell proliferation or p53. For instance, upregulation of NRG1, GPR87 and FGF2 is known to promote cell proliferation, whilst upregulation of GDF15, CDH10 and TNFRSF10C/TRAIL3 and downregulation of OTX1, CDCP1 and TCF7L1 are known to attenuate cell proliferation. Example p53 related genes that are reported to regulate or are regulated by p53 include MDM2, FDXR, GDF15, BBC3/PUMA and PPM1D/WIP1, some of whose function or gene expression changes in the lens are known (e.g., MDM2, GDF15, BBC3, FGF2, FAS). For more details, see S2 and S3 Tables and references are therein.

GO and KEGG pathways were analyzed for 5483 probes (3616 genes) that changed at *p* <0.05 (and FDR ≤1) at 3 h after 4 Gy vs 0 Gy, due to the limited number of genes that changed at *p* <0.05 and FDR <0.1. Hundreds of GO terms categorized in three domains were suggested at *p* <0.05 for up- or downregulated genes (S5 Table), among which the same 36 biological process, 3 molecular function and 29 cellular component terms were commonly suggested at *p* <0.001 for both up- and downregulated genes (these 68 terms listed in S6 Table). S2 Table shows 35 and 38 pathways suggested at *p* <0.05 each for up- and downregulated genes, of which 7 and 12 pathways were suggested at *p* <0.001, respectively (these 19 pathways listed in S7 Table). Pathways suggested at *p* <0.001 included p53 signaling for upregulated genes and cell cycle for downregulated genes (S7 Table). The same 7 pathways were commonly suggested at *p* <0.05 for both up- and downregulated genes (S8 Table), but there was no pathway suggested at *p* <0.001 (S5 Table).

To further examine whether pathways are suggested for activation or inhibition, the IPA analysis was performed for 1265 and 2234 genes that changed at *p* <0.0073 and <0.0126, respectively, at 3 h after 4 Gy vs 0 Gy. At these two *p* values, 22 canonical pathways, 23 diseases or functions annotations, and 13 upstream regulators were commonly suggested at z < –2 or >2 (highlighted with blue in S9–S11 Tables), many of which have been implicated in cataractogenesis or lenticular function, e.g., Rac, ephrin receptor and fibroblast growth factor (FGF) signaling in canonical pathways for activation [22–24], p53 and amyloid β precursor protein (APP) in upstream regulators for activation [25–27]. Many of diseases or functions annotations were related to cell proliferation and differentiation.

These results show that gene expression profiles in HLEC1 do not change at 3 or 8 h after 0.5 Gy irradiation, but that expression of some genes related to inactivation or stimulation of cell proliferation or p53 is changed at 3 h after 4 Gy irradiation.

### Conditioned medium does not alter proliferation nor clonogenicity in HLEC1

Finally, to test whether the autocrine or paracrine secretion of soluble pro- or antimitogenic factors from irradiated cells into culture medium affects cellular proliferative potential, we analyzed the impact of CM obtained at 13 days after sham or 2 Gy irradiation. S3 Fig shows that the 5 and 9 day treatment with CM does not change cell proliferation (panel A), and that the 14 day treatment with CM does not change the clonogenic potential (panel B). This suggests that CM does not change cell proliferation nor clonogenicity under conditions tested here.

## Discussion

Our previous study was the first to report the survival of irradiated lens cells, and found that HLEC1 contains subsets where irradiation (with dose of ≥2 Gy at a dose rate of 0.43 Gy/min) stimulates proliferation or facilitates abortive colony formation [11]. In turn, this study is the first to report premature senescence, gene expression changes analyzed with the DNA microarray analysis, and the impact of inhibition of DNA repair kinases following irradiation of lens cells, and obtained insights into the mechanisms behind our previous findings as discussed below.

### Implications for radiogenic inactivation and stimulation of HLEC1 proliferation

HLEC1 exhibited a growth delay without changing viability up to 7 days after *D*_10_ irradiation (Fig 1), and was more sensitive, especially at high CPD, to radiogenic premature senescence (evaluated here as SA-β-gal positivity) than WI-38 (Fig 2). Irreparable DNA damage induces permanent G_1_ arrest leading to premature senescence in a p53 dependent fashion [28]. The DNA microarray analysis at 3 h after 4 Gy vs 0 Gy suggested expression changes of various p53 related genes (S2 and S3 Tables). The KEGG pathway analysis suggested p53 signaling for upregulated genes (S7 Table), and the IPA analysis suggested p53 as an upstream regulator for activation (S11 Table). High sensitivity of HLEC1 to radiogenic premature senescence (Fig 3) accounts, at least in part, for its high sensitivity to radiogenic inactivation of clonogenic potential, formation of abortive colonies with less cells, and a growth delay without changing viability.

53BP1 foci in HLEC1 increased linearly with increasing dose at 0.5 h postirradiation (0.993< *r*^2^ <0.999 for the linearly fitted dose response curve at 0–1 Gy) (Fig 3). HLEC1 possessed less spontaneous foci but more radiogenic foci than WI-38 (Fig 3). Markiewicz et al. found a linear dose response for focus formation of phosphorylated histone H2AX (γH2AX) and RAD51, but not that of 53BP1, MRE11 and p53, at 1 h after X-irradiation of FHL124 (spontaneously immortalized human fetal LECs) with 0.14–2.28 Gy [29]. However, p53 accumulation that is generally known to begin within 0.5 h postirradiation [30] did not occur in FHL124 at 1 h after 0.14–2.28 Gy [29] indicative of impaired p53 function in FHL124. Also, high background levels of 53BP1, MRE11 and p53 immunofluorescence signals in sham-irradiated FHL124 [29] should have masked dose responses, in contrast to a low background of 53BP1 foci and its linear dose response in this study (Fig 3). Bannik et al. obtained LECs and lymphocytes from adult male C57BL/6J and JF1 mice, followed by *in vitro* irradiation [31]. LECs had less spontaneous foci and more radiogenic foci of γH2AX at 1 h after X-irradiation with 0–1 Gy, and 1 Gy-induced foci disappeared faster than lymphocytes in C57BL/6J [31], the former observation being akin to our findings for 53BP1 (Fig 3). Such differences between these two cell types in C57BL/6J, however, were less evident in JF1 [31], suggestive of strain dependence. Markiewicz et al. found a linear dose response for focus formation of γH2AX, RAD51 and 53BP1 occurring in LECs both in the central and peripheral regions of the lens epithelium at 1 h after *in vivo* X-irradiation of young male or female C57BL/6J mice with 0–0.1 Gy [29]. At 3 h postirradiation, disappearance of radiogenic γH2AX foci in the peripheral LECs was much slower than in the central LECs but was slightly faster than in lymphocytes [29], highlighting that DSB repair kinetics in LECs varies among regions within the lens epithelium. Wolf et al. found that most DNA strand breaks evaluated in LECs with the alkaline integrated comet assay are repaired within 30 min after *in vivo* irradiation of young female C57BL/6 mice, but that 8-OHG (oxidative DNA adduct) and XRCC1 (DNA single strand break repair protein) remain in the central and peripheral regions of the lens epithelium at 72 h postirradiation [32], suggesting the persistence of non-DSB damage in LECs following irradiation. These *in vitro* and *in vivo* findings are supportive of the present results for low frequency of spontaneous foci and high yield of radiogenic foci.

Treatment of HLEC1 with ATMi and DNA-PKi attenuated and delayed appearance and disappearance of 53BP1 foci, increased premature senescence, and enhanced clonogenic inactivation following irradiation (Figs 4–6), suggesting the involvement of ATM and DNA-PK. Intriguingly, it has been reported that mice hetero- or nullizygous for ATM are more prone to radiation cataracts than wild type counterparts [33], and the preliminary analysis of atomic bomb survivors has found a significant increase in cataract surgery prevalence in all minor homozygotes of three ATM haplotypes [34]. These highlight the important role of ATM in radiation cataracts, although such *in vivo* role of DNA-PK remains unknown.

The DNA microarray analysis in HLEC1 at 3 h after 4 Gy vs 0 Gy suggested expression changes of cell proliferation-related genes, such as upregulation of NRG1, GPR87 and FGF2 suggesting stimulation of cell proliferation, upregulation of GDF15, CDH10 and TNFRSF10C/TRAIL3 and downregulation of OTX1, CDCP1 and TCF7L1 suggesting attenuation of cell proliferation (S2 and S3 Tables). The IPA analysis (S9–S11 Tables) suggested the activation of FGF signaling (known to lead to LEC proliferation), and various other cell proliferation-related annotations (e.g., “generation of cells” with the highest z-score in S10 Table). These suggest both stimulation and attenuation of cell proliferation following irradiation, which explains at least partially our previous findings [11]. Taken together, expression changes of growth related genes including tumor suppressor genes p53, CDKN1B whose loss or accumulation increases cataracts [35,36], and PHLDA3 [37] are also interesting from the viewpoint of the implications of carcinogenesis related mechanisms for cataractogenesis [38]. All together, this encourages further studies to look at gene expression and other molecular changes separately in clonogenically stimulated cells and in clonogenially inactivated cells.

CM obtained at 13 days after plating of sham- and 2 Gy irradiated HLEC1 at cloning density (500 and 1500 cells/dish, respectively) did not affect cell proliferation nor clonogenicity (S2 Fig). The experimental condition employed here could mimic events taking place during colony formation, but the present negative result does not necessarily rule out the involvement of autocrine or paracrine mechanisms. Among genes upregulated at *p* <0.05 and FDR <0.1 at 3 h after 4 Gy vs 0 Gy, GDF15, IL1A, NRG1 and FGF2 are soluble, transmissible factors (Table 3). It will hence be interesting to test the impact of CM harvested under different conditions (e.g., CM harvested at earlier time points, CM harvested from cells irradiated with higher dose, and/or CM harvested from cells plated at higher density). Such analysis will also be encouraged to address the role of nontargeted effects in cataractogenesis [39–41].

### Other implications of observed gene expression changes

For the DNA microarray analysis, the dose point of 0.5 Gy was chosen because it is the new ICRP threshold for radiation cataracts [6]. No genes changed their expression levels at *p* <0.05 and FDR <0.1 at 3 or 8 h after 0.5 Gy vs 0 Gy (S1 Table). This was unexpected as 0.5 Gy is a dose that inactivates clonogenic potential of 31% of clonogenic HLEC1 [11], although expression changes at later time points need to be tested. The time and dose point of 3 h after 4 Gy was based on reports by Chang et al. who used pathway arrays and showed upregulation of FGF2 and CDKN1A and downregulation of cyclin G1, CDC2, CHK2, TIMP1, MMP-2, -3 and -9 at 3 h after irradiation of human LECs with 4 Gy of X-rays, protons, helium or iron ions [42–44]. Of these, upregulation of only FGF2 was observed in HLEC1 at *p* <0.05 and FDR <0.1, although upregulation of CDKN1B (p27^Kip1^), downregulation of CCNA2 (cyclin A2) and CCNB1 (cyclin B1) were observed instead (S3 and S4 Tables). Such a difference may stem from inconsistent experimental conditions such as: (*i*) whereas they used male LECs obtained from the 18-week prenatal lens that were cultured on extracellular matrix (ECM) derived from bovine corneal endothelial cells, we used female LECs obtained from the 24-week gestation fetal lens that were cultured without ECM; and (*ii*) whereas most of their findings come from particulate radiations (especially protons), we used only X-rays.

GDF15, THSD1, VWCE, SESN1, MDM2, BBC3, PCNA, FDXR, SESN2 and PPM1D were upregulated >1.5 fold at *p*<0.05 and FDR <0.1 at 3 h after 4 Gy vs 0 Gy (S3 Table), which have similarly been observed in other normal human cells (e.g., skin fibroblasts and blood cells) [45–47]. This suggests that these genes are common radioinducible genes in various types of normal human cells. Of these, there has been a growing effort for gene expression (trascriptomic) biodosimetry using FDXR, MDM2 and BBC3 as markers [48–50]. The present results support the usefulness of these markers in human lens cells at least at 4 Gy, and the dose response analysis is needed.

Various interesting suggestions were obtained from gene expression profiles evaluated with the DNA microarray analysis, so that further experiments are warranted to validate these results more quantitatively (e.g., with quantitative real time polymerase chain reaction) and to further examine expression changes at different dose and time points.

### Failure to immortalize HLEC1 with hTERT

Retrovirus-mediated transduction of hTERT did not prolong replicative lifespan of HLEC1 in contrast to the case of WI-38 (S1 Fig). The successful immortalization of primary human non-lens epithelial cells by retrovirus-mediated transduction of hTERT has been reported [51], but often requires co-transduction of an additional factor such as Bmi1, SV40 T/t antigens, p53 siRNA and RB siRNA [52–55]. Therefore, such co-transduction approach may be useful in HLEC1 as well. On the other hand, Huang et al. reported that electropolation of pCI-neo-hTERT extends replicative lifespan of primary adult human LECs [56]. As culture medium, they used DMEM with 20% FBS, and we used EpiCM. Replicative lifespan of HLEC1 was CPD 17.1 when cultured in EpiCM (S1 Fig) but was prolonged when cultured in DMEM with 20% FBS, suggesting that DMEM with 20% FBS is more mitogenic to HLEC1. Thus, DMEM with 20% FBS may help facilitate extension of lifespan or even induce transdifferentiation into myofibroblasts [57]. The establishment of human LEC cell line with the sustained ability for p53 function and for differentiation into lens fiber cells will be useful to clone and characterize a subset of clonogenically stimulated cells.

## Conclusions

Although the survival of irradiated HLEC1 was statistically indistinguishable from WI-38 [11], this study found several responses of HLEC1 different from WI-38. HLEC1 harbored less spontaneous foci and more radiogenic foci of 53BP1, and was more susceptible to radiogenic premature senescence than WI-38, which were dependent on ATM and DNA-PK. Changes in gene expression profiles suggested not merely the mechanisms for inhibition but also for stimulation of proliferation. Our present results partially explain mechanisms of our previous observations, such that unrepaired or incompletely repaired DNA damage causes a growth delay and formation of abortive colonies with less cells in a subset of HLEC1 without changing viability through induction of premature senescence, thereby culminating in clonogenic inactivation, but that growth is stimulated in another subset via as yet unidentified mechanisms. Further investigations are warranted to molecularly characterize a clonogenically stimulated subset and an inactivated subset, analyze changes in gene expression profiles at <4 Gy, and to examine the role of autocrine or paracrine mechanisms await further investigations.

## Supporting information

S1 Fig An attempt to prolong replicative lifespan by introduction of hTERT. (A) HLEC1 had *T*_D_ of 66.6 h at CPD 5.0–15.5 and ceased to divide at CPD 17.1. (B) HLEC1 infected at CPD 10.0 was serially passaged with weekly replenishments in the presence of G418. HLEC1/neo and HLEC1/hTERT ceased to divide at CPD 13.7 and 14.1, respectively. (C) WI-38 had *T*_D_ of 32.4 h at CPD 19.0–64.9 and ceased to divide at CPD 69.1. (D) WI-38 infected at CPD 31.0 was serially passaged with weekly replenishments in the presence of G418. WI-38/neo ceased to divide at CPD 51.6. WI-38/hTERT had *T*_D_ of 37.6 h at CPD 31.0–707 and continued to divide at least up to CPD 707. Panels (A) and (C) were taken from [11].
(TIFF)

S2 Fig Hierarchical cluster analysis. (A) A heatmap with a dendrogram added to the left side. (B) Gene expression pattern in each cluster. HLEC1 was subjected to RNA extraction at 3 h after irradiation with sham, 0.5 or 4 Gy, and at 8 h after irradiation with sham or 0.5 Gy. Then, the DNA microarray analysis was performed, and the results of hierarchical clustering are shown here. C1–C8 denote clusters 1–8. CPD at the time of plating was 11.5 ± 0.2, and dose rate was 0.44 ± 0.00 Gy/min. RNAs were obtained from three independent experiments.
(TIFF)

S3 Fig The impact of conditioned medium on cell proliferation and clonogenicity. (A) The number of HLEC1 treated for the indicated period with CM from 2 Gy irradiated cells relative to that from sham-irradiated cells, which was calculated each for days 5 and 9. (B) The plating efficiency of HLEC1 treated for 14 days during colony formation with CM from 2 Gy irradiated cells relative to that from sham-irradiated cells. Data are presented as means and SDs of two independent experiments with triplicate measurements.
(TIFF)

S1 Table Gene expression changes in X-irradiated HLEC1. (PDF)

S2 Table Genes whose expression changed in HLEC1 at *p* <0.05 and FDR <0.05 at 3 h after 4 Gy vs after 0 Gy. (PDF)

S3 Table Genes whose expression changed in HLEC1 >1.5 fold at *p* <0.05 and FDR <0.1 at 3 h after 4 Gy vs after 0 Gy. (PDF)

S4 Table Genes whose expression changed in HLEC1 <1.5 fold at *p* <0.05 and FDR <0.1 at 3 h after 4 Gy vs after 0 Gy. (PDF)

S5 Table Analysis of gene ontology and pathways in HLEC1 at 3 h after 4 Gy vs after 0 Gy. (PDF)

S6 Table Gene ontology terms suggested in HLEC1 at *p* <0.001 for both up- and downregulated genes at 3 h after 4 Gy vs after 0 Gy. (PDF)

S7 Table Pathways suggested in HLEC1 at *p* <0.001 at 3 h after 4 Gy vs after 0 Gy. (PDF)

S8 Table Pathways suggested in HLEC1 at *p* <0.05 for both up- and downregulated genes at 3 h after 4 Gy vs after 0 Gy. (PDF)

S9 Table Canonical pathways suggested in HLEC1 at 3 h after 4 Gy vs after 0 Gy. (PDF)

S10 Table Diseases or functions annotations suggested in HLEC1 at 3 h after 4 Gy vs after 0 Gy. (PDF)

S11 Table Upstream regulators suggested in HLEC1 at 3 h after 4 Gy vs after 0 Gy. (PDF)
