# Supplementary figures and images for: Ionizing radiation response of primary normal human lens epithelial cells
Source: PLoS One. 2017 Jul 26;12(7):e0181530. doi: 10.1371/journal.pone.0181530 (PMC5528879; doi:10.1371/journal.pone.0181530)

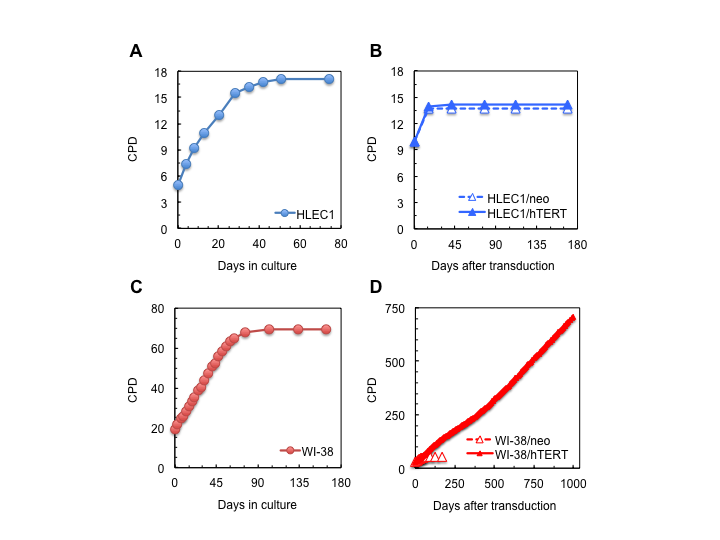

Supplement: S1 Fig — (A) HLEC1 had TD of 66.6 h at CPD 5.0–15.5 and ceased to divide at CPD 17.1. (B) HLEC1 infected at CPD 10.0 was serially passaged with weekly replenishments in the presence of G418. HLEC1/neo and HLEC1/hTERT ceased to divide at CPD 13.7 and 14.1, respectively. (C) WI-38 had TD of 32.4 h at CPD 19.0–64.9 and ceased to divide at CPD 69.1. (D) WI-38 infected at CPD 31.0 was serially passaged with weekly replenishments in the presence of G418. WI-38/neo ceased to divide at CPD 51.6. WI-38/hTERT had TD of 37.6 h at CPD 31.0–707 and continued to divide at least up to CPD 707. Panels (A) and (C) were taken from [11]. (TIFF) [file pone.0181530.s001.tiff]

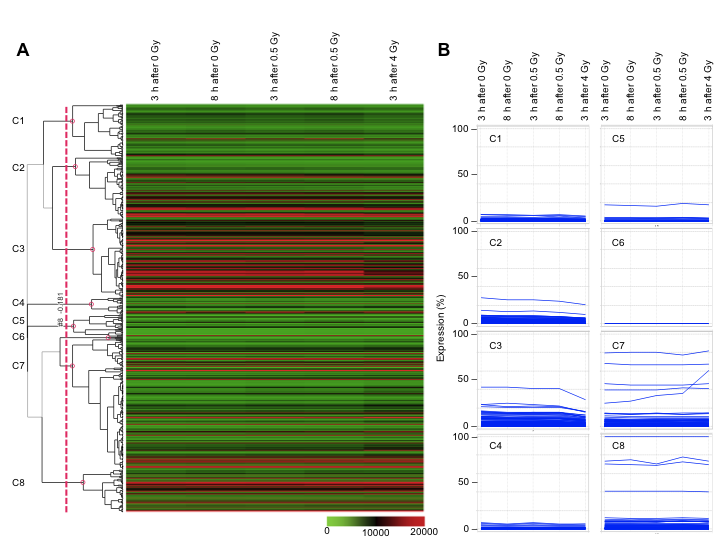

Supplement: S2 Fig — (A) A heatmap with a dendrogram added to the left side. (B) Gene expression pattern in each cluster. HLEC1 was subjected to RNA extraction at 3 h after irradiation with sham, 0.5 or 4 Gy, and at 8 h after irradiation with sham or 0.5 Gy. Then, the DNA microarray analysis was performed, and the results of hierarchical clustering are shown here. C1–C8 denote clusters 1–8. CPD at the time of plating was 11.5 ± 0.2, and dose rate was 0.44 ± 0.00 Gy/min. RNAs were obtained from three independent experiments. (TIFF) [file pone.0181530.s002.tiff]

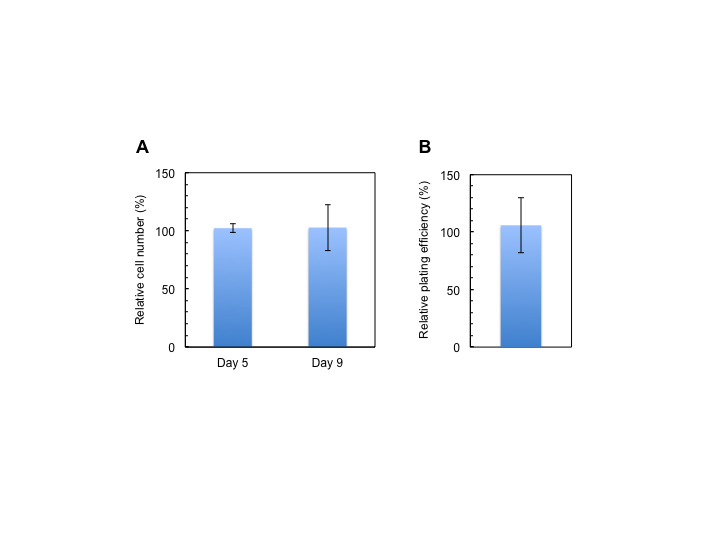

Supplement: S3 Fig — (A) The number of HLEC1 treated for the indicated period with CM from 2 Gy irradiated cells relative to that from sham-irradiated cells, which was calculated each for days 5 and 9. (B) The plating efficiency of HLEC1 treated for 14 days during colony formation with CM from 2 Gy irradiated cells relative to that from sham-irradiated cells. Data are presented as means and SDs of two independent experiments with triplicate measurements. (TIFF) [file pone.0181530.s003.tiff]
