# Supplementary material for: Ionizing radiation response of primary normal human lens epithelial cells
Source: PLoS One. 2017 Jul 26;12(7):e0181530. doi: 10.1371/journal.pone.0181530 (PMC5528879; doi:10.1371/journal.pone.0181530)
Supplement: S1 Table — (PDF) [file pone.0181530.s004.pdf]

S1 Table. Gene expression changes in X-irradiated HLEC1.

Comparison groups	p <0.05, FDR <0.05		p <0.05, FDR <0.1		p <0.05, FDR ≤1	
	Probes	Genes	Probes	Genes	Probes	Genes
8 h after 0 Gy vs 3 h after 0 Gy	0	0	0	0	2415	1688<br>↑ 843 (24[b])<br>↓ 845 (50[b])
8 h after 0.5 Gy vs 3 h after 0.5 Gy	0	0	0	0	3437	2231<br>↑ 1567 (156[b])<br>↓ 664 (24[b])
3 h after 0.5 Gy vs 3 h after 0 Gy	0	0	0	0	1894	1392<br>↑ 463 (15[b])<br>↓ 929 (66[b])
3 h after 4 Gy vs 3 h after 0 Gy	13	10[a]<br>↑ 6 (2[b])<br>↓ 4 (2[b])	456	356[c,d]<br>↑ 150 (22[b,e])<br>↓ 206 (13[b,e])	5483[f]	3616<br>↑ 1664 (96[b])<br>↓ 1952 (125[b])
8 h after 0.5 Gy vs 8 h after 0 Gy	0	0	0	0	2080	1376<br>↑ 833 (121[b])<br>↓ 543 (25[b])

FDR, false discovery rate. ↑, upregulation, ↓, downregulation. Information on the experimental condition is provided in the legends to S2 Fig.

[a] p <1.9 x 10^{-5} for all genes. All 10 genes are listed in S2 Table.

[b] The numer of genes with >1.5 fold changes.

[c] p <1.4 x 10^{-3} for all genes.

[d] All 321 genes with <1.5 fold changes are listed in S4 Table.

[e] All 35 genes with >1.5 fold changes are listed in S3 Table.

[f] The results of the pathway and gene ontology analysis are outlined in S5 Table.
