# Supplementary material for: Ionizing radiation response of primary normal human lens epithelial cells
Source: PLoS One. 2017 Jul 26;12(7):e0181530. doi: 10.1371/journal.pone.0181530 (PMC5528879; doi:10.1371/journal.pone.0181530)
Supplement: S2 Table — (PDF) [file pone.0181530.s005.pdf]

S2 Table. Genes whose expression changed in HLEC1 at *p* <0.05 and FDR <0.05 at 3 h after 4 Gy vs after 0 Gy.

Fold change	Gene symbol	Gene full name	GenBank accession number	Relation to p53	Growth-related or other functions
2.14	MDM2	MDM2 proto-oncogene	NM_002392	Yes	Upregulation increases growth [1S].<br>p53 regulation in the lens [2S]<br>LFC differentiation and organelle loss [3S].
1.74	FDXR	ferredoxin reductase	NM_004110	Yes	Upregulation does not alter growth [4S].
1.42	PHLDA3	pleckstrin homology-like domain family A member 3	NM_012396	Yes	Upregulation increases p53 accumulation [5S].
1.30	HMGN5	high mobility group nucleosome binding domain 5	NM_030763	N.A.	Upregulation increases growth [6S].<br>Downregulation radiosensitizes cells [7S].
1.17	VPS4B	vacuolar protein sorting 4 homolog B	NM_004869	N.A.	Downregulation decreases growth [8S].
1.10	GHITM	growth hormone inducible transmembrane protein	NM_014394	N.A.	Antiapoptotic [9S]
0.86	PPP4R1	protein phosphatase 4 regulatory subunit 1	NM_001042388	N.A.	Downregulation decreases growth [10S].
0.80	UBTD1	ubiquitin domain containing 1	NM_024954	Yes	Upregulation increases senescence [11S].
0.63	NFIX	nuclear factor I/X	NM_002501	N.A.	Upregulation decreases growth [12S].
0.63	PLEKHG5	pleckstrin homology and RhoGEF domain containing G5	NM_198681	N.A.	Downregulation decreases migration [13S].

FDR, false discovery rate. LFC, lens fiber cell. MDM2, murine double minute 2. N.A., not available. RhoGEF, rho-specific guanine nucleotide exchange factor. Information on the experimental condition is provided in the legends to S2 Fig. Pink and green areas highlight genes with >1.5 fold up- and downregulation, respectively.

References

1S. Huang Q, Hua HW, Jiang F, Liu DH, Ding G. Netrin-1 promoted pancreatic cancer cell proliferation by upregulation of Mdm2. Tumour Biol. 2014;35(10): 9927–9934. doi: 10.1007/s13277-014-2195-3. PMID: 25001177.

2S. Jaramillo-Rangel G, Ortega-Martínez M, Sepúlveda-Saavedra J, Saucedo-Cárdenas O, Montes-de-Oca-Luna R. p53 E3 ubiquitin protein ligase homolog regulates p53 in vivo in the adult mouse eye lens. Mol Vis. 2013;19:2468–2476. doi: unavailable. PMID: 24339722.

3S. Wride MA. Lens fibre cell differentiation and organelle loss: many paths lead to clarity. Philos Trans R Soc Lond B Biol Sci. 2011;366(1568):1219–1233. doi: 10.1098/rstb.2010.0324. PMID: 21402582.

4S. Liu G, Chen X. The ferredoxin reductase gene is regulated by the p53 family and sensitizes cells to oxidative stress-induced apoptosis. Oncogene. 2002;21(47):7195–7204. doi: 10.1038/sj.onc.1205862. PMID: 12370809.

5S. Lee CG, Kang YJ, Kim HS, Moon A, Kim SG. Phlda3, a urine-detectable protein, causes p53 accumulation in renal tubular cells injured by cisplatin. Cell Biol Toxicol. 2015;31(2):121–130. doi: 10.1007/s10565-015-9299-4. PMID: 25809501.

6S. Weng M, Song F, Chen J, Wu J, Qin J, Jin T, et al. The high-mobility group nucleosome-binding domain 5 is highly expressed in breast cancer and promotes the proliferation and invasion of breast cancer cells. Tumour Biol. 2015;36(2):959–966. doi: 10.1007/s13277-014-2715-1. PMID: 25315189.

7S. Su B, Shi B, Tang Y, Guo Z, Yu X, He X, et al. HMGN5 knockdown sensitizes prostate cancer cells to ionizing radiation. Prostate. 2015;75(1):33–44. doi: 10.1002/pros.22888. PMID: 25307178.

8S. Jiang D, Hu B, Wei L, Xiong Y, Wang G, Ni T, et al. High expression of vacuolar protein sorting 4B (VPS4B) is associated with accelerated cell proliferation and poor prognosis in human hepatocellular carcinoma. Pathol Res Pract. 2015;211(3):240–247. doi: 10.1016/j.prp.2014.11.013. PMID : 25547899.

9S. Reimers K, Choi CY, Bucan V, Vogt PM. The growth-hormone inducible transmembrane protein (Ghitm) belongs to the Bax inhibitory protein-like family. Int J Biol Sci. 2007;3(7):471–476. doi: 10.7150/ijbs.3.471. PMID: 18071587.

10S. Wu G, Ma Z, Qian J, Liu B. PP4R1 accelerates cell growth and proliferation in HepG2 hepatocellular carcinoma. Onco Targets Ther. 2015;8:2067–2074. doi: 10.2147/OTT.S77709. PMID: 26300649.

11S. Zhang XW, Wang XF, Ni SJ, Qin W, Zhao LQ, Hua RX, et al. UBTD1 induces cellular senescence through an UBTD1-Mdm2/p53 positive feedback loop. J Pathol. 2015;235(4):656–667. doi: 10.1002/path.4478. PMID: 25382750.

12S. Mao Y, Liu J, Zhang D, Li B. MiR-1290 promotes cancer progression by targeting nuclear factor I/X (NFIX) in esophageal squamous cell carcinoma (ESCC). Biomed Pharmacother. 2015;76:82–93. doi: 10.1016/j.biopha.2015.10.005. PMID: 26653554.

13S. Dachsel JC, Ngok SP, Lewis-Tuffin LJ, Kourtidis A, Geyer R, Johnston L, et al. The Rho guanine nucleotide exchange factor Syx regulates the balance of dia and ROCK activities to promote polarized-cancer-cell migration. Mol Cell Biol. 2013;33(24):4909–4918. doi: 10.1128/MCB.00565-13. PMID : 24126053.
