# Supplementary material for: Ionizing radiation response of primary normal human lens epithelial cells
Source: PLoS One. 2017 Jul 26;12(7):e0181530. doi: 10.1371/journal.pone.0181530 (PMC5528879; doi:10.1371/journal.pone.0181530)
Supplement: S3 Table — (PDF) [file pone.0181530.s006.pdf]

S3 Table. Genes whose expression changed in HLEC1 >1.5 fold at *p* <0.05 and FDR <0.1 at 3 h after 4 Gy vs after 0 Gy.

Fold change	Gene symbol	Gene full name	GenBank accession number	Relation to p53	Growth-related or other functions
2.86	GDF15	growth differentiation factor 15	NM_004864	Yes	Upregulation decreases growth [S14].<br>Upregulation increases radiogenic senescence [15S].<br>Upregulation after UVB in human LECs [16S]
2.69	IL1A	interleukin 1 alpha	NM_000575	N.A.	Proinflammatory cytokine
2.31	THSD1	thrombospondin type I domain containing 1	NM_018676	N.A.	Candidate tumor-suppressor gene [17S]
2.24	UNC5B-AS1	UNC5B antisense RNA 1	NR_038453	N.A.	N.A.
2.16	NRG1	neuregulin 1	AF176921	N.A.	Upregulation increases growth [18S].
2.15	VWCE	von Willebrand factor C and EGF domains	NM_152718	N.A.	N.A.
2.12	SESN1	sestrin 1	NM_014454	Yes	N.A.
2.08[a]	MDM2	MDM2 proto-oncogene	NM_002392	Yes	Upregulation increases growth [19S].<br>p53 regulation in the lens [20S]<br>LFC differentiation and organelle loss [21S]
2.07	GPR87	G protein-coupled receptor 87	NM_023915	Yes	Upregulation increases growth [22S].
2.07	BBC3[b]	BCL2 binding component 3	NM_014417	Yes	Upregulation increases growth [23S].<br>Proapoptotic [24S]<br>Downregulation causes radioresistance [24S].<br>Upregulation after UVB in mouse LECs [25S].
1.84[a]	PCNA	proliferating cell nuclear antigen	NM_002592	Yes	Upregulation increases growth [26S].<br>Downregulation decreases DNA repair [27S].
1.80	FAM212B	family with sequence similarity 212 member B	NM_019099	N.A.	Involved in migration [28S]
1.74	FDXR	ferredoxin reductase	NM_004110	Yes	Upregulation does not alter growth [29S].
1.72	CDH10	cadherin 10	NM_006727	N.A.	Upregulation decreases growth [30S].
1.72	SHC4	SHC adaptor protein 4	NM_203349	N.A.	Upregulation increases migration [31S].
1.67	REV3L	REV3 like, DNA directed polymerase zeta catalytic subunit	NM_002912	Yes	Downregulation decreases growth [32S].
1.67	SESN2	sestrin 2	NM_031459	Yes	Upregulation radiosensitizes cells [33S].
1.61	PPM1D[c]	protein phosphatase, Mg^{2+}/Mn^{2+} dependent 1D	NM_003620	Yes	Downregulation decreases growth [34S].<br>Upregulation radiosensitizes cells [35S].
1.58	TNFRSF10C	TNF receptor superfamily member 10c	NM_003841	Yes	Upregulation decreases growth [36S].
1.57	FGF2	fibroblast growth factor 2	NM_002006	Yes	Upregulation increases mouse LEC growth [37S].<br>Particles induce expression in human LECs [38S].
1.55	FAS	Fas cell surface death receptor	NM_000043	N.A.	Upregulation by GC stimulus in human LECs [39S].
1.52	WDR63	WD repeat domain 63	NM_145172	N.A.	N.A.
0.65	TRIM46	tripartite motif containing 46	NM_025058	N.A.	Downregulation decreases growth [40S].
0.64	ANKRD34A	ankyrin repeat domain 34A	NM_001039888	N.A.	N.A.
0.64	SOX5	SRY-box 5	NM_152989	N.A.	Downregulation decreases growth [41S].
0.64	TCF7L1	transcription factor 7-like 1	NM_031283	N.A.	Downregulation decreases growth [42S].
0.63	NFIX	nuclear factor I/X	NM_002501	N.A.	Upregulation decreases growth [43S].
0.63	SEPT6	septin 6	NM_145802	N.A.	N.A.
0.63	PLEKHG5	pleckstrin homology and RhoGEF domain containing G5	NM_198681	N.A.	Downregulation decreases migration [44S].
0.62[a]	CCNA2	cyclin A2	NM_001237	N.A.	Upregulation increases growth [45S].
0.62	CDCP1	CUB domain containing protein 1	NM_178181	N.A.	Downregulation decreases growth [46S].
0.61	PSRC1	proline and serine rich coiled-coil 1	NM_032636	Yes	N.A.
0.60	SPATA13	spermatogenesis associated 13	NM_153023	N.A.	Inhibition of migration [47S]
0.59	PBX3	PBX homeobox 3	NM_006195	N.A.	Upregulation increases growth [48S].
0.59	OTX1	orthodenticle homeobox 1	NM_014562	Yes	Downregulation decreases growth [49S].

BCL2, B cell leukemia/lymphoma 2. CUB, complement C1r/C1s, Uegf, Bmp1. EGF, epidermal growth factor. EMT, epithelial to mesenchymal transition. FDR, false discovery rate. GC, glucocorticoid. LEC, lens epithelial cell. LFC, lens fiber cell. MDM2, murine double minute 2. N.A., not available. PBX, pre-B-cell leukemia homeobox. RhoGEF, rho-specific guanine nucleotide exchange factor. SHC, Src homology 2 domain containing. SRY, sex determining region Y. TNF, tumor necrosis factor. UNC5B, uncoordinated-5 homolog netrin receptor B. UVB, ultraviolet B. Information on the experimental condition is provided in the legends to S2 Fig. Pink and green areas highlight genes with >1.5 fold up- and downregulation, respectively.

[a] Mean of 2–11 probes.

[b] Also known as PUMA (p53 upregulated mediator of apoptosis).

[c] Also known as WIP1 (wild-type p53-induced phosphatase 1).

References

14S. Tsui KH, Hsu SY, Chung LC, Lin YH, Feng TH, Lee TY, et al. Growth differentiation factor-15: a p53- and demethylation-upregulating gene represses cell proliferation, invasion, and tumorigenesis in bladder carcinoma cells. Sci Rep. 2015;5:12870. doi: 10.1038/srep12870. PMID: 26249737.

15S. Park H, Kim CH, Jeong JH, Park M, Kim KS. GDF15 contributes to radiation-induced senescence through the ROS-mediated p16 pathway in human endothelial cells. Oncotarget. 2016;7(9):9634–9644. doi: 10.18632/oncotarget.7457. PMID: 26909594.

16S. Osada H, Yoshitake Y, Ikeda T, Ishigaki Y, Takata T, Tomosugi N, et al. Ultraviolet B-induced expression of amphiregulin and growth differentiation factor 15 in human lens epithelial cells. Mol Vis. 2011;17:159–169. doi: unavailable. PMID: 21245963.

17S. Ko JM, Chan PL, Yau WL, Chan HK, Chan KC, Yu ZY, et al. Monochromosome transfer and microarray analysis identify a critical tumor-suppressive region mapping to chromosome 13q14 and THSD1 in esophageal carcinoma. Mol Cancer Res. 2008;6(4):592–603. doi: 10.1158/1541-7786.MCR-07-0154. PMID: 18403638.

18S. Umehara T, Kawashima I, Kawai T, Hoshino Y, Morohashi KI, Shima Y, et al. Endocrinology. 2016;157(12):4899–4913. doi: 10.1210/en.2016-1478. PMID: 27732090.

19S. Huang Q, Hua HW, Jiang F, Liu DH, Ding G. Netrin-1 promoted pancreatic cancer cell proliferation by upregulation of Mdm2. Tumour Biol. 2014;35(10):9927–9934. doi: 10.1007/s13277-014-2195-3. PMID: 25001177.

20S. Jaramillo-Rangel G, Ortega-Martínez M, Sepúlveda-Saavedra J, Saucedo-Cárdenas O, Montes-de-Oca-Luna R. p53 E3 ubiquitin protein ligase homolog regulates p53 in vivo in the adult mouse eye lens. Mol Vis. 2013;19:2468–2476. doi: unavailable. PMID: 24339722.

21S. Wride MA. Lens fibre cell differentiation and organelle loss: many paths lead to clarity. Philos Trans R Soc Lond B Biol Sci. 2011;366(1568):1219–1233. doi: 10.1098/rstb.2010.0324. PMID: 21402582.

22S. Zhang X, Liu D, Hayashida Y, Okazoe H, Hashimoto T, Ueda N, et al. G protein-coupled receptor 87 (GPR87) promotes cell proliferation in human bladder cancer cells. Int J Mol Sci. 2015;16(10):24319–24331. doi: 10.3390/ijms161024319. PMID: 26473854.

23S. Liu CJ, Zhang XL, Luo DY, Zhu WF, Wan HF, Yang JP, et al. Exogenous p53 upregulated modulator of apoptosis (PUMA) decreases growth of lung cancer A549 cells. Asian Pac J Cancer Prev. 2015;16(2):741–746. doi: unavailable. PMID: 25684518.

24S. Vávrová J, Rezáčová M. Importance of proapoptotic protein PUMA in cell radioresistance. Folia Biol (Praha). 2014;60(2):53–56. doi: unavailable. PMID : 24785107.

25S. Xiao F, Zhang JS, Zhao JY, Wu D. Regulation of Eaf2 in mouse lens cells apoptosis induced by ultraviolet radiation. Int J Ophthalmol. 2012;5(5):570–575. doi: 10.3980/j.issn.2222-3959.2012.05.05. PMID: 23166866.

26S. Juríková M, Danihel Ľ, Polák Š, Varga I. Ki67, PCNA, and MCM proteins: Markers of proliferation in the diagnosis of breast cancer. Acta Histochem. 2016;118(5):544–552. doi: 10.1016/j.acthis.2016.05.002. PMID: 27246286.

27S. Amorino GP, Mikkelsen RB, Valerie K, Schmidt-Ullrich RK. Dominant-negative cAMP-responsive element-binding protein inhibits proliferating cell

nuclear antigen and DNA repair, leading to increased cellular radiosensitivity. J Biol Chem. 2003;278(32):29394–29399. doi: 10.1074/jbc.M304012200. PMID: 12734192.
28S. Iwasaki Y, Yumoto T, Sakakibara S. Expression profiles of inka2 in the murine nervous system. Gene Expr Patterns. 2015;19(1–2):83–97. doi: 10.1016/j.gep.2015.08.002. PMID: 26292052.
29S. Liu G, Chen X. The ferredoxin reductase gene is regulated by the p53 family and sensitizes cells to oxidative stress-induced apoptosis. Oncogene. 2002;21(47):7195–7204. doi: 10.1038/sj.onc.1205862. PMID: 12370809.
30S. Li C, Gao Z, Li F, Li X, Sun Y, Wang M, et al. Whole exome sequencing identifies frequent somatic mutations in cell-cell adhesion genes in Chinese patients with lung squamous cell carcinoma. Sci Rep. 2015;5:14237. doi: 10.1038/srep14237. PMID: 26503331.
31S. Fagiani E, Giardina G, Luzi L, Cesaroni M, Quarto M, Capra M, et al. RaLP, a new member of the Src homology and collagen family, regulates cell migration and tumor growth of metastatic melanomas. Cancer Res. 2007;67(7):3064–3073. doi: 10.1158/0008-5472.CAN-06-2301. PMID: 17409413.
32S. Lange SS, Wittschieben JP, Wood RD. DNA polymerase zeta is required for proliferation of normal mammalian cells. Nucleic Acids Res. 2012;40(10): 4473–4482. doi: 10.1093/nar/gks054. PMID: 22319213.
33S. Sanli T, Linher-Melville K, Tsakiridis T, Singh G. Sestrin2 modulates AMPK subunit expression and its response to ionizing radiation in breast cancer cells. PLoS One. 2012;7(2):e32035. doi: 10.1371/journal.pone.0032035. PMID: 22363791.
34S. Zhang C, Chen Y, Wang M, Chen X, Li Y, Song E, et al. PPM1D silencing by RNA interference inhibits the proliferation of lung cancer cells. World J Surg Oncol. 2014;12:258. doi: 10.1186/1477-7819-12-258. PMID: 25123458.
35S. Pechackova S, Burdova K, Benada J, Kleiblova P, Jenikova G, Macurek L. Inhibition of WIP1 phosphatase sensitizes breast cancer cells to genotoxic stress and to MDM2 antagonist nutlin-3. Oncotarget. 2016;7(12):14458–14475. doi: 10.18632/oncotarget.7363. PMID: 26883108.
36S. Sriraksa R, Limpaiboon T. TRAIL in combination with subtoxic 5-FU effectively inhibit cell proliferation and induce apoptosis in cholangiocarcinoma cells. Asian Pac J Cancer Prev. 2015;16(16):6991–6996. doi: unavailable. PMID: 26514480.
37S. Tanaka T, Saika S, Ohnishi Y, Ooshima A, McAvoy JW, Liu CY, et al. Fibroblast growth factor 2: roles of regulation of lens cell proliferation and epithelial-mesenchymal transition in response to injury. Mol Vis. 2004;10:462–467. doi: unavailable. PMID: 15273655.
38S. Chang PY, Bjornstad KA, Chang E, McNamara M, Barcellos-Hoff MH, Lin SP, et al. Particle irradiation induces FGF2 expression in normal human lens cells. Radiat Res. 2000;154(5):477–484. doi: unavailable. PMID: 11025644.
39S. Zhou D, Zhang Y, Wang L, Sun Y, Liu P. Identification of genes and transcription factors associated with glucocorticoid response in lens epithelial cells. Mol Med Rep. 2015;11(6):4073–4078. doi: 10.3892/mmr.2015.3308. PMID: 25672806.
40S. Zhang L, Li X, Dong W, Sun C, Guo D, Zhang L. Mmu-miR-1894-3p inhibits cell proliferation and migration of breast cancer cells by targeting Trim46. Int J Mol Sci. 2016;17(4):609. doi: 10.3390/ijms17040609. PMID: 27110773.
41S. Pei XH, Lv XQ, Li HX. Sox5 induces epithelial to mesenchymal transition by transactivation of Twist1. Biochem Biophys Res Commun. 2014;446(1): 322–327. doi: 10.1016/j.bbrc.2014.02.109. PMID: 24607904.
42S. Ma H, Mallampati S, Lu Y, Sun B, Wang E, Leng X, et al. The Sox4/Tcf7l1 axis promotes progression of BCR-ABL-positive acute lymphoblastic leukemia. Haematologica. 2014;99(10):1591–1598. doi: 10.3324/haematol.2014.104695. PMID: 24997151.
43S. Mao Y, Liu J, Zhang D, Li B. MiR-1290 promotes cancer progression by targeting nuclear factor I/X (NFIX) in esophageal squamous cell carcinoma (ESCC). Biomed Pharmacother. 2015;76:82–93. doi: 10.1016/j.biopha.2015.10.005. PMID: 26653554.
44S. Dachsel JC, Ngok SP, Lewis-Tuffin LJ, Kourtidis A, Geyer R, Johnston L, et al. The Rho guanine nucleotide exchange factor Syx regulates the balance of dia and ROCK activities to promote polarized-cancer-cell migration. Mol Cell Biol. 2013;33(24):4909–4918. doi: 10.1128/MCB.00565-13. PMID : 24126053.
45S. Lee CT, Boeshore KL, Wu C, Becker KG, Errico SL, Mash DC, et al. Cocaine promotes primary human astrocyte proliferation via JNK-dependent up-regulation of cyclin A2. Restor Neurol Neurosci. 2016;34(6):965–976. doi: 10.3233/RNN-160676. PMID: 27834787.
46S. Harrington BS, He Y, Davies CM, Wallace SJ, Adams MN, Beaven EA, et al. Cell line and patient-derived xenograft models reveal elevated CDCP1 as a target in high-grade serous ovarian cancer. Br J Cancer. 2016;114(4):417–426. doi: 10.1038/bjc.2015.471. PMID: 26882065.
47S. Jean L, Yang L, Majumdar D, Gao Y, Shi M, Brewer BM, et al. The Rho family GEF Asef2 regulates cell migration in three dimensional (3D) collagen matrices through myosin II. Cell Adh Migr. 2014;8(5):460–467. doi: 10.4161/19336918.2014.983778. PMID: 25517435.
48S. Li Y, Sun Z, Zhu Z, Zhang J, Sun X, Xu H. PBX3 is overexpressed in gastric cancer and regulates cell proliferation. Tumour Biol. 2014;35(5):4363–4368. doi: 10.1007/s13277-013-1573-6. PMID: 24375258.
49S. Li H, Miao Q, Xu CW, Huang JH, Zhou YF, Wu MJ. OTX1 contributes to hepatocellular carcinoma progression by regulation of ERK/MAPK pathway. J Korean Med Sci. 2016;31(8):1215–1223. doi: 10.3346/jkms.2016.31.8.1215. PMID: 27478331.
