# Supplementary material for: Ionizing radiation response of primary normal human lens epithelial cells
Source: PLoS One. 2017 Jul 26;12(7):e0181530. doi: 10.1371/journal.pone.0181530 (PMC5528879; doi:10.1371/journal.pone.0181530)
Supplement: S4 Table — (PDF) [file pone.0181530.s007.pdf]

S4 Table. Genes whose expression changed in HLEC1 <1.5 fold at *p* <0.05 and FDR <0.1 at 3 h after 4 Gy vs after 0 Gy.

Fold change	Gene symbol	Gene full name	GenBank Accession number
1.48	TMEM68	transmembrane protein 68	NM_152417
1.47	EDA2R	ectodysplasin A2 receptor	NM_001242310
1.46	PHLDB3	pleckstrin homology like domain family B member 3	NM_198850
1.42	PDGFA	platelet derived growth factor subunit A	NM_002607
1.42	ANKRA2	ankyrin repeat family A member 2	NM_023039
1.42	PHLDA3	pleckstrin homology like domain family A member 3	NM_012396
1.40	SLC40A1	solute carrier family 40 member 1	NM_014585
1.39	TRAF4	TNF receptor associated factor 4	NM_004295
1.37	CKB	creatine kinase B	NM_001823
1.36	SUSD6	sushi domain containing 6	NM_014734
1.35	TRIAP1	TP53 regulated inhibitor of apoptosis 1	NM_016399
1.34	PHF1	PHD finger protein 1	NM_024165
1.34	RCBTB1	RCC1 and BTB domain containing protein 1	NM_018191
1.33	DKK1	dickkopf WNT signaling pathway inhibitor 1	NM_012242
1.33	SPTY2D1	SPT2 chromatin protein domain containing 1	NM_194285
1.33	DDB2	damage specific DNA binding protein 2	NM_000107
1.32	STXBP3	syntaxin binding protein 3	NM_007269
1.32	POLH	DNA polymerase eta	NM_006502
1.31	SLC4A11	solute carrier family 4 member 11	NM_032034
1.31	LINC00963	long intergenic non-protein coding RNA 963	NR_038955
1.29	CDKN1B	cyclin dependent kinase inhibitor 1B	NM_004064
1.29	FAM198B	family with sequence similarity 198 member B	NM_016613
1.29	GADD45A	growth arrest and DNA damage inducible alpha	NM_001924
1.28	GNAI1	G protein subunit alpha i1	NM_002069
1.28	DCP1B	decapping mRNA 1B	NM_152640
1.27[a]	HMGN5	high mobility group nucleosome binding domain 5	NM_030763
1.27	IKBIP	IKBKB interacting protein	NM_201612
1.27	ENAH	enabled homolog (Drosophila)	NM_001008493
1.26	SLC19A2	solute carrier family 19 member 2	NM_006996
1.26	FZD5	frizzled class receptor 5	NM_003468
1.25	MGC27345	uncharacterized protein MGC27345	NR_046216
1.25	ZNF182	zinc finger protein 182	NM_006962
1.25	NR3C1	nuclear receptor subfamily 3 group C member 1	NM_001018077
1.25[a]	CCDC59	coiled-coil domain containing 59	NM_014167
1.24	RGL1	Ral guanine nucleotide dissociation stimulator like 1	NM_015149
1.24	RALGAPB	Ral GTPase activating protein non-catalytic beta subunit	NM_020336
1.24	HIST2H2AB	histone cluster 2 H2A family member b	NM_175065
1.24	ARL8B	ADP ribosylation factor like GTPase 8B	NM_018184
1.24	XPC	XPC complex subunit, DNA damage recognition and repair factor	NM_004628
1.24	VPS37D	VPS37D, ESCRT-I subunit	NM_001077621
1.23[a]	PTEN	phosphatase and tensin homolog	NM_000314
1.22	MPDZ	multiple PDZ domain crumbs cell polarity complex component	NM_003829
1.22	TAF13	TATA-box binding protein associated factor 13	NM_005645
1.22	C2CD3	C2 calcium dependent domain containing 3	NM_015531
1.21	MRPL39	mitochondrial ribosomal protein L39	NM_017446
1.21	ATP13A3	ATPase type 13A3	NM_024524
1.21	KLHL20	kelch like family member 20	NM_014458
1.21	EML4	echinoderm microtubule associated protein like 4	NM_019063
1.21	BZW1	basic leucine zipper and W2 domains 1	NM_014670
1.21	KIAA0368	KIAA0368	NM_001080398
1.21	ISOC1	isochorismatase domain containing 1	NM_016048
1.20	CETN3	centrin 3	NM_004365
1.20[a]	ERO1L	endoplasmic reticulum oxidoreductase 1 alpha	NM_014584
1.20	TMEM9B	TMEM9 domain family member B	NM_020644
1.20	SNRPF	small nuclear ribonucleoprotein polypeptide F	NM_003095
1.20	PRKCD	protein kinase C, delta	NM_006254
1.19	RFK	riboflavin kinase	NM_018339
1.19	SLC41A2	solute carrier family 41 member 2	NM_032148
1.19	DNAJC10	DnaJ heat shock protein family (Hsp40) member C10	NM_018981
1.19	TMEM127	transmembrane protein 127	NM_017849
1.19	RBM41	RNA binding motif protein 41	AK023732
1.18	IQCJ-SCHIP1	IQCJ-SCHIP1 readthrough	NM_001197113
1.18	HDHD2	haloacid dehalogenase like hydrolase domain containing 2	NM_032124
1.18	CAPRIN1	cell cycle associated protein 1	NM_005898
1.18	TRAM1	translocation associated membrane protein 1	NM_014294
1.18	SMARCAD1	SWI/SNF-related, matrix-associated actin-dependent regulator of chromatin, subfamily a, containing DEAD/H box 1	NM_001128429
1.17	EIF1B	eukaryotic translation initiation factor 1B	NM_005875
1.17	NDUFC2	NADH:ubiquinone oxidoreductase subunit C2	NM_004549
1.17	VPS4B	vacuolar protein sorting 4 homolog B	NM_004869
1.17	EIF4A2	eukaryotic translation initiation factor 4A2	NM_001967
1.17	NARS	asparaginyl-tRNA synthetase	NM_004539
1.17	CLASP2	cytoplasmic linker associated protein 2	NM_015097
1.16	RNF11	ring finger protein 11	NM_014372
1.16	BOLA3	bolA family member 3	NM_212552
1.16	MZT1	mitotic spindle organizing protein 1	NM_001071775
1.16	MRPS36	mitochondrial ribosomal protein S36	NM_033281
1.15	CPSF4	cleavage and polyadenylation specific factor 4	NM_006693
1.15	DDX5	DEAD-box helicase 5	NM_004396
1.15	BBS2	Bardet-Biedl syndrome 2	NM_031885
1.15	SRSF5	serine and arginine rich splicing factor 5	NM_001039465
1.15	SARNP	SAP domain containing ribonucleoprotein	NM_033082
1.15	RYK	receptor-like tyrosine kinase	NM_001005861
1.15	SEC24B	SEC24 homolog B, COPII coat complex component	NM_006323
1.15	COA6	cytochrome c oxidase assembly factor 6	NM_001012985
1.15	WDR26	WD repeat domain 26	NM_025160

Fold change	Gene symbol	Gene full name	GenBank Accession number
1.15	CCDC6	coiled-coil domain containing 6	NM_005436
1.15	RPL26	ribosomal protein L26	NM_000987
1.15	G3BP2	G3BP stress granule assembly factor 2	NM_203505
1.15[a]	FAM102A	family with sequence similarity 102 member A	NM_001035254
1.14	BTBD1	BTB domain containing 1	NM_025238
1.14	SNHG12	small nucleolar RNA host gene 12	NR_024127
1.14	SERTAD1	SERTA domain containing 1	NM_013376
1.14	TRAPPC2B	trafficking protein particle complex 2B	NR_002166
1.14	TOP2B	topoisomerase (DNA) II beta	NM_001068
1.14	NMI	N-myc and STAT interactor	NM_004688
1.14	SLU7	SLU7 homolog, splicing factor	NM_006425
1.13	GDI1	GDP dissociation inhibitor 1	NM_001493
1.13[a]	RB1	RB transcriptional corepressor 1	NM_000321
1.13[a]	RPL30	ribosomal protein L30	NM_000989
1.13	SNX6	sorting nexin 6	NM_021249
1.13	GNG10	G protein subunit gamma 10	NM_001017998
1.13	ORMDL1	ORMDL sphingolipid biosynthesis regulator 1	NM_016467
1.13	SEPT10	septin 10	NM_144710
1.13	SNHG6	small nucleolar RNA host gene 6	NR_002599
1.12	RPL31	ribosomal protein L31	NM_000993
1.12	NACC2	NACC family member 2	NM_144653
1.12	TPT1	tumor protein, translationally-controlled 1	NM_003295
1.12	MOB4	MOB family member 4, phocein	NM_015387
1.12	SKIV2L2	Ski2 like RNA helicase 2	NM_015360
1.12	RWDD1	RWD domain containing 1	NM_016104
1.11	NDUFS6	NADH:ubiquinone oxidoreductase subunit S6	NM_004553
1.11	RBMS1	RNA binding motif single stranded interacting protein 1	NM_016836
1.10	R3HDM2	R3H domain containing 2	NM_014925
1.10	PROCR	protein C receptor	NM_006404
1.10	GHITM	growth hormone inducible transmembrane protein	NM_014394
1.10	SUCO	SUN domain containing ossification factor	NM_016227
1.10	TVP23C	trans-Golgi network vesicle protein 23 homolog C (S. cerevisiae)	NM_145301
1.09	RNF25	ring finger protein 25	NM_022453
1.09	PERP	PERP, TP53 apoptosis effector	NM_022121
1.09	ZNHIT3	zinc finger HIT-type containing 3	NM_004773
1.08	MARVELD1	MARVEL domain containing 1	NM_031484
1.08	TFB2M	transcription factor B2, mitochondrial	NM_022366
1.08	KDELC1	KDEL motif containing 1	NM_024089
1.08	YWHAB	tyrosine 3-monooxygenase/tryptophan 5-monooxygenase activation protein beta	NM_003404
1.07	LAMTOR5	late endosomal/lysosomal adaptor, MAPK and MTOR activator 5	NM_006402
1.07	OPTN	optineurin	NM_001008211
1.07	ARPC2	actin related protein 2/3 complex subunit 2	NM_152862
1.06	COPS9	COP9 signalosome subunit 9	NM_001163424
0.96	IARS	isoleucyl-tRNA synthetase	NM_013417
0.95	DTD1	D-tyrosyl-tRNA deacylase 1	NM_080820
0.94	TMEM132A	transmembrane protein 132A	NM_017870
0.94	XXYLT1	xyloside xylosyltransferase 1	NM_152531
0.92	DECR2	2,4-dienoyl CoA reductase 2, peroxisomal	NM_020664
0.92	NDUFS7	NADH:ubiquinone oxidoreductase core subunit S7	NM_024407
0.92	EIF2B3	eukaryotic translation initiation factor 2B subunit 3 gamma	NM_020365
0.91	PABPN1	poly(A) binding protein nuclear 1	NM_004643
0.91	MPHOSPH8	M-phase phosphoprotein 8	NM_017520
0.91	CNOT10	CCR4-NOT transcription complex subunit 10	NM_015442
0.91	AP5S1	adaptor related protein complex 5 sigma 1 subunit	NM_018347
0.91	PC	pyruvate carboxylase	NM_001040716
0.91	PGLS	6-phosphogluconolactonase	NM_012088
0.90	CKS2	CDC28 protein kinase regulatory subunit 2	NM_001827
0.90	MAPK1	mitogen-activated protein kinase 1	NM_138957
0.90	KLF16	Kruppel like factor 16	NM_031918
0.90	RARB	retinoic acid receptor beta	NM_000965
0.89	TFIP11	tuftelin interacting protein 11	NM_001008697
0.89	TST	thiosulfate sulfurtransferase	NM_003312
0.89	KANK1	KN motif and ankyrin repeat domains 1	NM_153186
0.89	ZNF783	zinc finger family member 783	NM_001195220
0.89	GMPS	guanine monphosphate synthetase	NM_003875
0.88	DDX28	DEAD-box helicase 28	NM_018380
0.88	SPATS2	spermatogenesis associated serine-rich 2	NM_023071
0.88	CREBBP	CREB binding protein	NM_004380
0.88	TBCD	tubulin folding cofactor D	NM_005993
0.88[a]	VPS72	vacuolar protein sorting 72 homolog	NM_005997
0.88	OR7E24	olfactory receptor family 7 subfamily E member 24	NM_001079935
0.88	ISY1	ISY1 splicing factor homolog	NM_020701
0.88	SIPA1	signal-induced proliferation-associated 1	NM_153253
0.88	DVL1	dishevelled segment polarity protein 1	NM_004421
0.88	MTND4L	mitochondrially encoded NADH 4L dehydrogenase	AK311996
0.88	RNF168	ring finger protein 168	NM_152617
0.88	NCLN	nicalin	NM_020170
0.87	MIPEP	mitochondrial intermediate peptidase	NM_005932
0.87	ABHD14A	abhydrolase domain containing 14A	NM_015407
0.87	EVA1A	eva-1 homolog A, regulator of programmed cell death	NM_032181
0.87	SPATA2L	spermatogenesis associated 2 like	NM_152339
0.87	LOC100506459	uncharacterized LOC100506459	XR_110492[b]
0.87	LAMB2	laminin subunit beta 2	NM_002292
0.87	CC2D2A	coiled-coil and C2 domain containing 2A	NM_001080522
0.86	TMPRSS6	transmembrane protease, serine 6	NM_153609
0.86	SLC2A6	solute carrier family 2 member 6	NM_017585

Fold change	Gene symbol	Gene full name	GenBank Accession number
0.86	RPA1	replication protein A1	NM_002945
0.86	SMOX	spermine oxidase	NM_175839
0.86	GPATCH4	G patch domain containing 4	NM_182679
0.86	MAP7D3	MAP7 domain containing 3	NM_024597
0.86	BPNT1	3'(2'), 5'-bisphosphate nucleotidase 1	NM_006085
0.86	COMMD5	COMM domain containing 5	NM_014066
0.86	PPP4R1	protein phosphatase 4 regulatory subunit 1	NM_001042388
0.86	LRRC20	leucine rich repeat containing 20	NM_018205
0.85	CRYL1	crystallin lambda 1	NM_015974
0.85	BAD	BCL2 associated agonist of cell death	NM_004322
0.85	IQSEC2	IQ motif and Sec7 domain 2	NM_015075
0.85	EXOC7	exocyst complex component 7	NM_001145297
0.85	MSTO1	misato 1, mitochondrial distribution and morphology regulator	NM_018116
0.85	GGA3	Golgi associated, gamma adaptin ear containing, ARF binding protein 3	NM_138619
0.85	CPT1C	carnitine palmitoyltransferase 1C	NM_001199752
0.85	MON1A	MON1 homolog A, secretory trafficking associated	NM_032355
0.85	TRAF2	TNF receptor associated factor 2	NM_021138
0.85	MEF2B	myocyte enhancer factor 2B	NM_001145785
0.85	GTF2IRD1	GTF2I repeat domain containing 1	NM_005685
0.85	RAB36	RAB36, member RAS oncogene family	NM_004914
0.85	MARCH4	membrane associated ring-CH-type finger 4	NM_020814
0.85	FBXO17	F-box protein 17	NM_024907
0.85	VARS	valyl-tRNA synthetase	NM_006295
0.85[a]	SRRT	serrate, RNA effector molecule	NM_015908
0.84	EIF2B5	eukaryotic translation initiation factor 2B subunit epsilon	NM_003907
0.84	BCS1L	BCS1 homolog, ubiquinol-cytochrome c reductase complex chaperone	NM_004328
0.84	SYNGR1	synaptogyrin 1	NM_145731
0.84	BCAT2	branched chain amino acid transaminase 2	NM_001190
0.84	NOB1	NIN1/PSMD8 binding protein 1 homolog	NM_014062
0.84	SPRY4	sprouty RTK signaling antagonist 4	NM_030964
0.84	APBA1	amyloid beta precursor protein binding family A member 1	NM_001163
0.84	SMARCC2	SWI/SNF related, matrix associated, actin dependent regulator of chromatin subfamily c member 2	NM_139067
0.84	ZNF628	zinc finger protein 628	NM_033113
0.84	TAF6	TATA-box binding protein associated factor 6	NM_005641
0.83	ETV6	ets variant 6	NM_001987
0.83	LONP2	lon peptidase 2, peroxisomal	NM_031490
0.83	SMARCAL1	SWI/SNF related, matrix associated, actin dependent regulator of chromatin, subfamily a like 1	NM_014140
0.83	CCDC86	coiled-coil domain containing 86	NM_024098
0.83	HYLS1	HYLS1, centriolar and ciliogenesis associated	NM_145014
0.83	FICD	FIC domain containing	NM_007076
0.83	SUSD2	sushi domain containing 2	NM_019601
0.83	WIPI2	WD repeat domain, phosphoinositide interacting 2	NM_001033518
0.83	XPO5	exportin 5	NM_020750
0.82	NCSTN	nicastrin	NM_015331
0.82	PLIN2	perilipin 2	NM_001122
0.82	DHX37	DEAH-box helicase 37	NM_032656
0.82	CCDC106	coiled-coil domain containing 106	NM_013301
0.82	DHX34	DEAH-box helicase 34	NM_014681
0.82	GNB5	G protein subunit beta 5	NM_016194
0.82	CENPP	centromere protein P	NM_001012267
0.82	SHMT1	serine hydroxymethyltransferase 1	NM_004169
0.82	KAT2A	lysine acetyltransferase 2A	NM_021078
0.82	YY1AP1	YY1 associated protein 1	NM_139118
0.82	SCPEP1	serine carboxypeptidase 1	NM_021626
0.82	CTDSP1	CTD small phosphatase 1	NM_182642
0.81	ETNK2	ethanolamine kinase 2	NM_018208
0.81	NOL12	nucleolar protein 12	NM_024313
0.81	NSUN5	NOP2/Sun RNA methyltransferase family member 5	NM_148956
0.81[a]	WDR24	WD repeat domain 24	AK301146
0.81	KRTAP3-3	keratin associated protein 3-3	NM_033185
0.81	TRIOBP	TRIO and F-actin binding protein	NM_001039141
0.81	POLR3H	RNA polymerase III subunit H	NM_138338
0.81	TCAF1	TRPM8 channel associated factor 1	NM_014719
0.81	DESI2	desumoylating isopeptidase 2	NM_016076
0.81	SMARCD3	SWI/SNF related, matrix associated, actin dependent regulator of chromatin, subfamily d, member 3	NM_003078
0.81	CARM1	coactivator associated arginine methyltransferase 1	NM_199141
0.81	RNPEPL1	arginyl aminopeptidase like 1	NM_018226
0.81	KRTAP2-3	keratin associated protein 2-3	NM_001165252
0.81	APRT	adenine phosphoribosyltransferase	NM_000485
0.81	POLRMT	RNA polymerase mitochondrial	NM_005035
0.81	MGC4859	uncharacterized LOC79150	BC002644
0.81	FMR1	fragile X mental retardation 1	NM_002024
0.81	CHCHD2	coiled-coil-helix-coiled-coil-helix domain containing 2	NM_016139
0.80	RBFOX2	RNA binding protein, fox-1 homolog 2	NM_001031695
0.80	STX1A	syntaxin 1A	NM_004603
0.80	PRPSAP1	phosphoribosyl pyrophosphate synthetase associated protein 1	NM_002766
0.80	PARP2	poly(ADP-ribose) polymerase 2	NM_005484
0.80	ALKBH2	alkB homolog 2, alpha-ketoglutarate dependent dioxygenase	NM_001001655
0.80	COA7	cytochrome c oxidase assembly factor 7 (putative)	NM_023077
0.80	CTU1	cytosolic thiouridylase subunit 1	NM_145232
0.80	RUFY1	RUN and FYVE domain containing 1	NM_025158
0.80	UBTD1	ubiquitin domain containing 1	NM_024954
0.80	RNFT2	ring finger protein, transmembrane 2	NM_032814

Fold change	Gene symbol	Gene full name	GenBank Accession number
0.80[a]	BBX	BBX, HMG-box containing	NM_020235
0.80	TESK1	testis-specific kinase 1	NM_006285
0.80	C1QTNF6	C1q and tumor necrosis factor related protein 6	NM_031910
0.79	TMEM37	transmembrane protein 37	NM_183240
0.79	LOC728743	zinc finger protein pseudogene	NR_027237
0.79	MEX3A	mex-3 RNA binding family member A	NM_001093725
0.79	RNF31	ring finger protein 31	NM_017999
0.79[a]	ATAD3B	ATPase family, AAA domain containing 3B	NM_031921
0.79	AGAP1	ArfGAP with GTPase domain, ankyrin repeat and PH domain 1	NM_001037131
0.79	TMEM223	transmembrane protein 223	NM_001080501
0.78	MRPL42P5	mitochondrial ribosomal protein L42 pseudogene 5	NR_002208
0.78	ZNF395	zinc finger protein 395	NM_018660
0.78	NDUFV1	NADH:ubiquinone oxidoreductase core subunit V1	NM_007103
0.78[a]	RABEP2	rabaptin, RAB GTPase binding effector protein 2	NM_024816
0.78	ASMTL	acetylserotonin O-methyltransferase-like	NM_004192
0.78[a]	CPTP	ceramide-1-phosphate transfer protein	NM_001029885
0.77	PPRC1	peroxisome proliferator-activated receptor gamma, coactivator-related 1	NM_015062
0.77	ZBED3	zinc finger BED-type containing 3	NM_032367
0.77	KIAA1549L	KIAA1549 like	NM_012194
0.77	LRP5L	LDL receptor related protein 5 like	NM_182492
0.77	ATAD3A	ATPase family, AAA domain containing 3A	NM_018188
0.76	CTXN1	cortexin 1	NM_206833
0.76	ZNF444	zinc finger protein 444	NM_018337
0.76	ASB13	ankyrin repeat and SOCS box containing 13	NM_024701
0.76	RPTOR	regulatory associated protein of MTOR, complex 1	NM_020761
0.76	POLE3	DNA polymerase epsilon 3, accessory subunit	NM_017443
0.76	CCNB1	cyclin B1	NM_031966
0.76	SLC45A3	solute carrier family 45 member 3	NM_033102
0.75	FAM83G	family with sequence similarity 83, member G	NM_001039999
0.75[a]	GFOD1	glucose-fructose oxidoreductase domain containing 1	NM_018988
0.75	ZNRF3	zinc and ring finger 3	NM_001206998
0.75	EVI5L	ecotropic viral integration site 5 like	NM_145245
0.75	DDX31	DEAD-box helicase 31	NM_138620
0.75	LOC644189	acyl-CoA thioesterase 4 pseudogene	NR_033748
0.74	SBNO2	strawberry notch homolog 2	NM_014963
0.74	SLX4	SLX4 structure-specific endonuclease subunit	NM_032444
0.74	CBX2	chromobox 2	NM_005189
0.73	F8A1	coagulation factor VIII-associated 1	NM_012151
0.73	PEX11B	peroxisomal biogenesis factor 11 beta	NM_003846
0.73	LINC00857	long intergenic non-protein coding RNA 857	NR_038464
0.73	PLEKHM1	pleckstrin homology and RUN domain containing M1	NM_014798
0.73	ZNF587	zinc finger protein 587	BC011243
0.73	PARP10	poly(ADP-ribose) polymerase family member 10	NM_032789
0.72	E2F5	E2F transcription factor 5	NM_001951
0.72	PIF1	PIF1 5'-to-3' DNA helicase	NM_025049
0.72	TFEB	transcription factor EB	NM_007162
0.72	TRO	trophinin	NM_016157
0.72	REEP6	receptor accessory protein 6	NM_138393
0.71	INCENP	inner centromere protein	NM_001040694
0.71	TMEM175	transmembrane protein 175	NM_032326
0.71	LAMTOR4	late endosomal/lysosomal adaptor, MAPK and MTOR activator 4	NM_001008395
0.71	MAP3K11	mitogen-activated protein kinase kinase kinase 11	NM_002419
0.71	DLGAP4	DLG associated protein 4	NM_014902
0.71	ZBTB47	zinc finger and BTB domain containing 47	NM_145166
0.71	TYW1	tRNA-yW synthesizing protein 1 homolog	NM_018264
0.70	DCBLD1	discoidin, CUB and LCCL domain containing 1	NM_173674
0.70	GGA1	Golgi-associated, gamma adaptin ear containing, ARF binding protein 1	NM_001001560
0.70	ADD1	adducin 1	NM_014189
0.70	FAM26E	family with sequence similarity 26 member E	NM_153711
0.69	SLC43A2	solute carrier family 43 member 2	NM_152346
0.69	MXD4	MAX dimerization protein 4	NM_006454
0.69	FBXL15	F-box and leucine rich repeat protein 15	NM_024326
0.68	HIP1	huntingtin interacting protein 1	NM_005338
0.67[a]	TBL1Y	transducin beta like 1, Y-linked	NM_033284
0.67	TBL1X	transducin beta like 1X-linked	NM_005647
0.67	BEST1	bestrophin 1	NM_004183
0.67	KDM5B	lysine demethylase 5B	NM_006618

AAA, ATPases associated with diverse cellular activities. AlkB, alpha-ketoglutarate-dependent dioxygenase B. ARF, alternative reading frame. ArfGAP, ADP ribosylation factor GTPase activating protein. BBX, bobby sox. BED, boundary element-associated factor and DNA replication-related element factor. BTB, broad complex, tramtrack and bric-à-brac. CCR4-NOT, carbon catabolite repression 4 - negative on TATA-less. CDC28, cell division control 28. COMM, copper metabolism MURR1 (mouse U2af1-rs1 region 1). COP9, constitutive photomorphogenesis protein 9. COPII, coat protein complex II. CREB, cAMP-response element binding protein. CTD, carboxy-terminal domain. CUB, C1r/C1s, Uegf and Bmp1. DEAD, Asp-Glu-Ala-Asp. DEAD/H, Asp-Glu-Ala-Asp/His. DEAH, Asp-Glu-Ala-His. DLG, discs large. ESCRT-I, endosomal sorting complexes required for transport I. ets, E26. eva-1, enhancer of unc-40 ventral axon guidance defects. FDR, false discovery rate. FIC, filamentation induced by cAMP. Fox-1, feminizing gene on X 1. FYVE, Fab1, YOTB/ZK632.12, Vac1, and EEA1. GTF2I, general transcription factor IIi. GSBP, GTPase-activating protein-(Src homology 3 domain)-binding protein. HMG box, high mobility group box. HYLS1, hydrolethalus syndrome 1. IKBKB, inhibitor of kappaB kinase beta. IQCJ-SCHIP1, IQ motif containing J and schwannomin interacting protein 1. ISY1, interactor of Syf1p. KDEL, Lys-Asp-Glu-Leu. KN motif, Kank N-terminal motif. LCCL, Limulus clotting factor C, cochlear protein Coch-5b2 and late gestation lung protein Lgl1. LDL, low density lipoprotein. MAP7, microtubule-associated protein 7. MAPK, mitogen-activated protein kinase. MARVEL, MAL and related proteins for vesicle trafficking and membrane link. mex-3, muscle excess protein-3. MOB, Mps one binder. mTOR, mammalian target of rapamycin. NADH, β-nicotinamide adenine dinucleotide reduced. NOP2, nucleolar protein 2. ORMDL, ORM1 like. PDZ, post synaptic density protein, Drosophila disc large tumor suppressor, and zonula occludens-1. PERP, p53 apoptosis effector related to PMP-22. PH domain, pleckstrin homology domain. PHD, plant homeodomain. PSMD8, proteasome 26S subunit, non-ATPase 8. RB, retinoblastoma. RCC1, regulator of chromosome condensation 1. RTK, receptor tyrosine kinase. RUN, RPIP8, unc-14 and NESCA. RWD, RING finger-containing proteins, WD-repeat-containing proteins, and yeast DEAD (DEXD)-like helicases. R3H, an invariant arginine and a highly conserved histidine separated by three residues. SAP, scaffold attachment factors A and B, acinus and protein inhibitor of activated STAT. SERTA, SEI-1, RBT1 and TARA. Ski2, superkiller 2. SLU7, pre-mRNA splicing regulator. SOCS, suppressor of cytokines signaling. SPT2, suppressors of Ty insertions gene family 2. SUN, Sad1 and UNC-84. SWI/SNF, switch/sucrose non-fermenting. TMEM9,

transmemberane protein 9. TNF, tumor necrosis factor. tRNA, transfer RNA. TRPM8, transient receptor potential melastatin 8. VPS37D, vacuolar protein sorting 37 homolog D. WD repeat, Trp-Asp repeat. XPC, xeroderma pigmentosum complementation group C. yW, wybutosine. YY1, Yin-Yang 1. Information on the experimental condition is provided in the legends to S2 Fig.

[a] Mean of 2–6 probes.

[b] Record removed as a result of standard genome annotation processing.
