# Supplementary material for: Ionizing radiation response of primary normal human lens epithelial cells
Source: PLoS One. 2017 Jul 26;12(7):e0181530. doi: 10.1371/journal.pone.0181530 (PMC5528879; doi:10.1371/journal.pone.0181530)
Supplement: S5 Table — (PDF) [file pone.0181530.s008.pdf]

S5 Table. Analysis of gene ontology and pathways in HLEC1 at 3 h after 4 Gy vs after 0 Gy.

			Gene ontology			Pathway
			Biological process	Molecular function	Cellular component	
Probes						
	Total		5483	5483	5483	5483
	Upregulation		2661	2661	2661	2661
	Downregulation		2822	2822	2822	2822
Genes						
	Total		2802	2866	3046	3616
	Upregulation		1331	1348	1423	619
	Downregulation		1471	1518	1623	621
Suggested pathways or gene ontology terms						
	Upregulation					
		Total (*p* <0.05)	585	129	145	35
		p <0.001	156	15	58	7[b]
		0.001≤ *p* <0.01	124	32	25	10
		0.01≤ *p* <0.05	305	82	62	18
	Downregulation					
		Total (*p* <0.05)	387	109	104	38
		p <0.001	79	12	44	12[b]
		0.001≤ *p* <0.01	97	34	23	7
		0.01≤ *p* <0.05	211	63	37	19
	Overlap between up- and downregulation					
		Total (*p* <0.05)	132	15	48	7[c]
		p <0.001	36[a]	3[a]	29[a]	0

Information on the experimental condition is provided in the legends to S2 Fig.

[a] All 68 gene ontology terms are listed in S6 Table.

[b] All 19 suggested pathways are listed in S7 Table.

[c] All 7 suggested pathways are listed in S8 Table.
