# Supplementary material for: Ionizing radiation response of primary normal human lens epithelial cells
Source: PLoS One. 2017 Jul 26;12(7):e0181530. doi: 10.1371/journal.pone.0181530 (PMC5528879; doi:10.1371/journal.pone.0181530)
Supplement: S6 Table — (PDF) [file pone.0181530.s009.pdf]

S6 Table. Gene ontology terms suggested in HLEC1 at *p* <0.001 for both up- and downregulated genes at 3 h after 4 Gy vs after 0 Gy.

Gene ontology domain	Gene ontology term name[a]	Gene ontology term number[b]	*p* values	
			Upregulated genes	Downregulated genes
Biological process	Cellular biopolymer metabolic process	GO:0034960	2.5×10^{-15}	9.1×10^{-14}
	Cellular protein metabolic process	GO:0044267	3.6×10^{-14}	7.0×10^{-5}
	Cellular macromolecule metabolic process	GO:0044260	1.5×10^{-14}	1.6×10^{-12}
	Cellular metabolic process	GO:0044237	2.2×10^{-13}	3.9×10^{-11}
	Macromolecule metabolic process	GO:0043170	4.8×10^{-12}	1.6×10^{-9}
	Biopolymer metabolic process	GO:0043283	1.1×10^{-11}	5.1×10^{-11}
	Nucleobase, nucleoside, nucleotide and nucleic acid metabolic process	GO:0006139	9.0×10^{-10}	1.9×10^{-6}
	Metabolic process	GO:0008152	2.6×10^{-10}	2.1×10^{-8}
	Nitrogen compound metabolic process	GO:0006807	6.6×10^{-9}	4.4×10^{-7}
	Gene expression	GO:0010467	2.9×10^{-9}	1.0×10^{-5}
	Cellular component organization	GO:0016043	6.0×10^{-8}	1.2×10^{-5}
	Primary metabolic process	GO:0044238	3.0×10^{-8}	3.5×10^{-9}
	Macromolecule biosynthetic process	GO:0009059	2.6×10^{-7}	3.9×10^{-6}
	Cellular macromolecule biosynthetic process	GO:0034645	2.0×10^{-7}	4.7×10^{-6}
	Biopolymer biosynthetic process	GO:0043284	1.8×10^{-7}	2.0×10^{-6}
	RNA metabolic process	GO:0016070	1.5×10^{-7}	3.1×10^{-6}
	Cellular biopolymer biosynthetic process	GO:0034961	1.3×10^{-7}	2.4×10^{-6}
	Biosynthetic process	GO:0009058	9.9×10^{-6}	2.6×10^{-4}
	Cellular biosynthetic process	GO:0044249	7.7×10^{-6}	2.3×10^{-4}
	Organelle organization	GO:0006996	1.3×10^{-6}	4.2×10^{-8}
	DNA metabolic process	GO:0006259	9.0×10^{-5}	2.5×10^{-4}
	Biopolymer modification	GO:0043412	1.6×10^{-5}	6.2×10^{-4}
	Cell cycle	GO:0007049	1.4×10^{-5}	3.1×10^{-6}
	Regulation of cell cycle	GO:0051726	1.2×10^{-5}	9.6×10^{-4}
	Regulation of primary metabolic process	GO:0080090	9.3×10^{-4}	1.2×10^{-4}
	Regulation of cellular metabolic process	GO:0031323	5.6×10^{-4}	4.5×10^{-7}
	RNA biosynthetic process	GO:0032774	4.8×10^{-4}	7.0×10^{-5}
	Regulation of metabolic process	GO:0019222	4.7×10^{-4}	3.6×10^{-6}
	Transcription, DNA-dependent	GO:0006351	4.5×10^{-4}	8.3×10^{-5}
	Cellular component biogenesis	GO:0044085	4.4×10^{-4}	2.5×10^{-4}
	Chromatin organization	GO:0006325	3.8×10^{-4}	6.1×10^{-4}
	Cellular process	GO:0009987	3.4×10^{-4}	3.0×10^{-7}
	Chromatin modification	GO:0016568	2.6×10^{-4}	4.7×10^{-5}
	Transcription	GO:0006350	1.6×10^{-4}	6.7×10^{-5}
	Regulation of macromolecule metabolic process	GO:0060255	1.1×10^{-4}	2.5×10^{-6}
	Chromosome organization	GO:0051276	1.1×10^{-4}	2.6×10^{-5}
Molecular function	Protein binding	GO:0005515	8.0×10^{-10}	6.7×10^{-6}
	Binding	GO:0005488	7.9×10^{-8}	2.4×10^{-8}
	Nucleic acid binding	GO:0003676	3.9×10^{-7}	6.6×10^{-5}
Cellular component	Intracellular organelle	GO:0043229	2.7×10^{-37}	4.2×10^{-24}
	Organelle	GO:0043226	2.0×10^{-36}	2.3×10^{-23}
	Intracellular part	GO:0044424	1.0×10^{-34}	2.0×10^{-35}
	Intracellular	GO:0005622	1.0×10^{-33}	2.5×10^{-34}
	Intracellular membrane-bounded organelle	GO:0043231	7.4×10^{-30}	1.4×10^{-23}
	Membrane-bounded organelle	GO:0043227	4.7×10^{-29}	4.9×10^{-23}
	Organelle part	GO:0044422	2.1×10^{-23}	2.7×10^{-22}
	Intracellular organelle part	GO:0044446	1.7×10^{-23}	1.0×10^{-22}
	Cytoplasm	GO:0005737	4.8×10^{-21}	2.5×10^{-14}
	Cytoplasmic part	GO:0044444	9.4×10^{-20}	1.7×10^{-9}
	Cytosol	GO:0005829	4.5×10^{-15}	7.6×10^{-8}
	Nucleus	GO:0005634	3.0×10^{-15}	9.0×10^{-20}
	Macromolecular complex	GO:0032991	1.0×10^{-14}	7.8×10^{-6}
	Nucleoplasm	GO:0005654	2.6×10^{-11}	1.5×10^{-12}
	Membrane-enclosed lumen	GO:0031974	4.3×10^{-10}	4.1×10^{-19}
	Intracellular organelle lumen	GO:0070013	3.9×10^{-10}	7.7×10^{-19}
	Cell	GO:0005623	3.8×10^{-10}	7.9×10^{-9}
	Nuclear lumen	GO:0031981	3.8×10^{-10}	7.5×10^{-18}
	Cell part	GO:0044464	3.6×10^{-10}	7.5×10^{-9}
	Nuclear part	GO:0044428	1.3×10^{-9}	3.6×10^{-18}
	Organelle lumen	GO:0043233	1.1×10^{-9}	2.8×10^{-18}
	Non-membrane-bounded organelle	GO:0043228	2.7×10^{-8}	1.7×10^{-10}
	Intracellular non-membrane-bounded organelle	GO:0043232	2.7×10^{-8}	1.7×10^{-10}
	Nucleoplasm part	GO:0044451	2.4×10^{-7}	5.0×10^{-8}
	Protein complex	GO:0043234	8.9×10^{-6}	4.3×10^{-6}
	Mitochondrion	GO:0005739	7.7×10^{-5}	3.2×10^{-5}
	Nucleolus	GO:0005730	1.7×10^{-4}	1.4×10^{-7}
	Nuclear body	GO:0016604	1.5×10^{-4}	6.7×10^{-4}
	Chromosome	GO:0005694	1.4×10^{-4}	8.6×10^{-4}

Information on the experimental condition is provided in the legends to S2 Fig.

[a] For each gene ontology domain, gene ontology terms with lower *p* values for upregulated genes appear in the upper rows.

[b] The information on gene ontology terms is available at http://amigo.geneontology.org/cgi-bin/amigo/term_details?term=GO:00xxxxx e.g., for "Cellular biopolymer metabolic process", at http://amigo.geneontology.org/cgi-bin/amigo/term_details?term=GO:0034960.
