# Supplementary material for: Ionizing radiation response of primary normal human lens epithelial cells
Source: PLoS One. 2017 Jul 26;12(7):e0181530. doi: 10.1371/journal.pone.0181530 (PMC5528879; doi:10.1371/journal.pone.0181530)
Supplement: S7 Table — (PDF) [file pone.0181530.s010.pdf]

S7 Table. Pathways suggested in HLEC1 at *p* <0.001 at 3 h after 4 Gy vs after 0 Gy.

Pathway name	Pathway map number[a]	*p* values
Suggested for upregulated genes		
Ribosome	hsa03010	3.4×10^{-13}
Spliceosome	hsa03040	5.3×10^{-6}
p53 signaling pathway	hsa04115	6.7×10^{-6}
Huntington's disease	hsa05016	1.1×10^{-4}
Parkinson's disease	hsa05012	3.0×10^{-4}
Oxidative phosphorylation	hsa00190	3.8×10^{-4}
FoxO signaling pathway	hsa04068	9.1×10^{-4}
Suggested for downregulated genes		
Adherens junction	hsa04520	2.2×10^{-5}
Cell cycle	hsa04110	4.6×10^{-5}
Pathways in cancer	hsa05200	1.0×10^{-4}
Non-small cell lung cancer	hsa05223	1.1×10^{-4}
Metabolic pathways	hsa01100	2.0×10^{-4}
Notch signaling pathway	hsa04330	2.8×10^{-4}
Epstein-Barr virus infection	hsa05169	3.9×10^{-4}
Neurotrophin signaling pathway	hsa04722	5.0×10^{-4}
Homologous recombination	hsa03440	6.2×10^{-4}
Pancreatic cancer	hsa05212	7.7×10^{-4}
Fanconi anemia pathway	hsa03460	7.9×10^{-4}
Thyroid cancer	hsa05216	8.3×10^{-4}

Information on the experimental condition is provided in the legends to S2 Fig.

[a] Maps are available at http://www.kegg.jp/kegg-bin/show_pathway?map=hsa0xxxx
e.g., for "Ribosome", at http://www.kegg.jp/kegg-bin/show_pathway?map=hsa03010.
