# Supplementary material for: Ionizing radiation response of primary normal human lens epithelial cells
Source: PLoS One. 2017 Jul 26;12(7):e0181530. doi: 10.1371/journal.pone.0181530 (PMC5528879; doi:10.1371/journal.pone.0181530)
Supplement: S8 Table — (PDF) [file pone.0181530.s011.pdf]

S8 Table. Pathways suggested in HLEC1 at p <0.05 for both up- and downregulated genes at 3 h after 4 Gy vs after 0 Gy.

Pathway name[a]	Pathway map number[b]	p values[c]	
		Upregulated genes	Downregulated genes
Prostate cancer	hsa05215	0.0010	0.0010
Notch signaling pathway	hsa04330	0.0104	0.0003
Epstein-Barr virus infection	hsa05169	0.0182	0.0004
Proteoglycans in cancer	hsa05205	0.0230	0.0239
Glioma	hsa05214	0.0329	0.0143
Pathways in cancer	hsa05200	0.0350	0.0001
Viral carcinogenesis	hsa05203	0.0362	0.0374

Information on the experimental condition is provided in the legends to S2 Fig.

[a] Pathways with lower p values for upregulated genes appear in the upper rows.

[b] Maps are available at http://www.kegg.jp/kegg-bin/show_pathway?map=hsa0xxxx
e.g., for "Prostate cancer", at http://www.kegg.jp/kegg-bin/show_pathway?map=hsa05215.

[c] No common pathways were suggested at p <0.001 for both up- and downregulated genes.
