# Supplementary material for: Ionizing radiation response of primary normal human lens epithelial cells
Source: PLoS One. 2017 Jul 26;12(7):e0181530. doi: 10.1371/journal.pone.0181530 (PMC5528879; doi:10.1371/journal.pone.0181530)
Supplement: S9 Table — (PDF) [file pone.0181530.s012.pdf]

S9 Table. Canonical pathways suggested in HLEC1 at 3 h after 4 Gy vs after 0 Gy.

Canonical pathways[a]	z-score[b]	Genes
iCOS-iCOSL signaling in T helper cells	3.0	PTPRC,LCK,CD40LG,HLA-DQA1,TRAT1,FGFR2,IL2RA,KLB,HLA-DRB5
Dendritic cell maturation	3.0	CD1D,CD40LG,HLA-DQA1,FGFR2,IGHG1,IL23A,KLB,HLA-DRB5,FCGR1B
Signaling by Rho family GTPases	3.0	GNAS,CDH10,GNAO1,FGFR2,PAK5,GFAP,PIP5K1B,KLB,ARHGEF10,NOX1
Colorectal cancer metastasis signaling	2.9	MMP27,TLR10,GNAS,PROK1,DCC,WNT16,WNT4,FGFR2,VEGFD,HNF1A,KLB,WNT2
CREB signaling in neurons	2.8	GRIN2B,GRIN2A,GNAS,GRID1,GRIK3,GNAO1,FGFR2,GRIK2,KLB
Role of NFAT in regulation of the immune response	2.8	LCK,GNAS,GNAO1,HLA-DQA1,FGFR2,KLB,HLA-DRB5,FCGR1B
Rac signaling	2.6	MCF2L,ABI2,FGFR2,PAK5,PIP5K1B,KLB,NOX1
ILK signaling	2.6	MYH4,PROK1,FGFR2,VEGFD,RPS6KA5,MYH11,KLB
cAMP-mediated signaling	2.5	ENPP6,P2RY14,GNAS,CNGA4,HTR1E,GLP1R,GNAO1,TAAR1,HRH3,PDE6H
Retinoic acid mediated apoptosis signaling	2.4	RXRG,PARP15,IFNA8,IFNW1,ART1,IFNK,CRABP1,PARP1
Relaxin signaling	2.4	ENPP6,GNAS,GUCY2C,GUCY2D,GNAO1,GUCY2F,FGFR2,KLB,PDE6H
UVA-induced MAPK signaling	2.4	PARP15,ART1,FGFR2,RPS6KA5,KLB,PARP1
Role of pattern recognition receptors in recognition of bacteria and viruses	2.4	MBL2,IFNA8,IFNW1,FGFR2,IFNK,KLB
Tec kinase signaling	2.4	LCK,GNAS,GNAO1,FGFR2,PAK5,KLB,FAS
Synaptic long term depression	2.4	GNAS,GUCY2C,GUCY2D,GRID1,GNAO1,GUCY2F
Gαq signaling	2.4	HTR2C,GNAS,FGFR2,AVPR1A,KLB,HTR2A
Glutamate receptor signaling	2.2	GRIN2B,GRIN2A,SLC1A6,SLC17A6,GRID1,SLC17A2,GRIK3,GRIK2
Ephrin receptor signaling	2.2	EPHA6,EPHA10,GRIN2B,EPHB1,GRIN2A,GNAS,PROK1,GNAO1,VEGFD,PAK5,EPHA3
Basal cell carcinoma signaling	2.2	BMP8B,WNT16,WNT4,HNF1A,WNT2
Role of PI3K/AKT signaling in the pathogenesis of influenza	2.2	IFNA8,IFNW1,FGFR2,IFNK,KLB
FGF signaling	2.2	FGF2,HGF,FGFR2,RPS6KA5,KLB
PAK signaling	2.2	FGFR2,PAK5,DSCAM,EPHA3,KLB
Th1 pathway	2.2	CD40LG,KLRD1,HLA-DQA1,FGFR2,KLB,HLA-DRB5
Corticotropin releasing hormone signaling	2.2	GNAS,GUCY2C,GUCY2D,GNAO1,GUCY2F
PKCθ signaling in T lymphocytes	2.2	LCK,HLA-DQA1,FGFR2,KLB,HLA-DRB5
Cardiac β-adrenergic signaling	2.2	ENPP6,GNAS,PPP1R1A,ATP2A3,PDE6H
IL-8 signaling	2.1	GNAS,FLT1,PROK1,FGFR2,VEGFD,KLB,NOX1,CR2
Melanocyte development and pigmentation signaling	2.0	GNAS,TYR,PAX3,FGFR2,RPS6KA5,KLB
PEDF signaling	2.0	GDNF,FGFR2,HNF1A,KLB,FAS
IL-2 signaling	2.0	LCK,FGFR2,IL2RA,KLB
Calcium-induced T lymphocyte apoptosis	2.0	LCK,HLA-DQA1,ATP2A3,HLA-DRB5
TREM1 signaling	2.0	TLR10,NLRP6,IL1RL1,CIITA
CD28 signaling in T helper cells	2.0	PTPRC,LCK,HLA-DQA1,FGFR2,KLB,HLA-DRB5
Renal cell carcinoma signaling	2.0	HGF,FGFR2,PAK5,KLB
Acute myeloid leukemia signaling	2.0	FLT3,FGFR2,HNF1A,KLB
Death receptor signaling	2.0	PARP15,ART1,FAS,PARP1
Mouse embryonic stem cell pluripotency	2.0	FGFR2,FOXD3,HNF1A,KLB
Telomerase signaling	2.0	FGFR2,IL2RA,ELF5,KLB

Information on the experimental condition is provided in the legends to S2 Fig.

[a] Analyses were conducted for 1265 genes that changed at p <0.0073. Blue area highlights pathways that also yielded the z-score of >2 for 2234 genes whose expression changed at p <0.0126.

[b] Canonical pathways with the z-score of >2 (indicative of activation) or < –2 (indicative of inhibition) are listed. No pathways with z < –2 were suggested.
