# Supplementary material for: Ionizing radiation response of primary normal human lens epithelial cells
Source: PLoS One. 2017 Jul 26;12(7):e0181530. doi: 10.1371/journal.pone.0181530 (PMC5528879; doi:10.1371/journal.pone.0181530)
Supplement: S10 Table — (PDF) [file pone.0181530.s013.pdf]

S10 Table. Diseases or functions annotations suggested in HLEC1 at 3 h after 4 Gy vs after 0 Gy.

Categories[a]	Diseases or functions annotation	z-score[b]	Genes
Cellular growth and proliferation, tissue development	Generation of cells	5.5	ABI2,ADGRB3,ALOX5,ASIC2,ASZ1,ATOH1,ATOH7,AVPR1A,BBC3,BTG2,CAMP, CAPZA3,CBLN1,CD1D,CD40LG,CFAP54,CIITA,CNTN2,CR2,CRISP2,CSF3, CTNNA2,CUX2,CXCL11,DAZ2,DCC,DCX,DPYSL5,DSCAM,EBF3,EPHA3,EPHB1, ERG,FAS,FGF2,FGFR2,FLT1,FLT3,FSHB,GALNTL5,GDNF,GFAP,GNAO1,HAND1, HGF,HLA-DQA1,HNF1A,HOXA13,IFNL3,IGF2,IGHG1,IKZF1,IL1RL1,IL20RA,IL23A, IL2RA,KCNJ2,KDF1,KISS1R,LCK,LHX6,LRAT,LRRC7,LRRTM4,MAS1,MBP,MEI4, MSR1,MUC2,MYH11,MYO16,NCKAP1L,NKX2-8,NLGN3,NPPA,NRG1,NTRK3, OLFM3,OTX2,PARP1,PAX3,PAX5,PBX3,PCDHB6,PDILT,PGR,PIP5K1B,PLG, POU2AF1,PRDM14,PRLR,PROK1,PROK2,PTPRC,RBMY1A1 (includes others), RIMS1,RIPK3,RIT2,RPGRIP1,RTN3,RXRG,S100B,SHANK2,SIT1,SLAMF1, SLC17A6,SLC5A7,SLIT1,SLITRK3,SOX5,SPATA22,ST8SIA2,TFAP2A,TFAP4, THEMIS,TMEM106B,TRAT1,TSSK2,UBR2,VEGFD,VPREB1,VWC2,WFDC2,WNT2, WNT4,XRCC5,ZC3H13
Cell signaling, molecular transport, vitamin and mineral metabolism	Mobilization of Ca^{2+}	4.2	ALOX12,AVPR1A,CAMP,CCL23,CCL8,CR2,CX3CR1,CXCL11,CXCL17,DIO2, FCRL5,GNAO1,HTR2A,HTR2C,IGHG1,KISS1R,LCK,MAS1,MLNR,PKHD1, PPP1R1B,PROK2,PTPRC,SRL,TPSD1,TRAT1,WNT4,XCL1
Nervous system development and function, tissue morphology	Quantity of neurons	3.8	ATOH1,ATOH7,CNTN2,CUX2,CX3CR1,DCC,DSCAM,FGF2,FGFR2,GAD1,GDF15, GDNF,GNAO1,GNAS,GRID1,GRIN2B,HGF,KISS1R,LHX6,MDM2,NKX2-8,NLGN3, NRG1,NTRK3,OTX2,RIMS1,SLC17A6,ST8SIA2,TBR1,TFAP2A,VAX1,WNT4,XRCC5
Nervous system development and function, tissue morphology	Quantity of nervous tissue	3.6	ATOH1,ATOH7,CNTN2,CUX2,CX3CR1,DCC,DSCAM,FGF2,FGFR2,GAD1,GDF15, GDNF,GNAO1,GNAS,GRID1,GRIN2B,HGF,KISS1R,LHX6,MDM2,NKX2-8,NLGN3, NRG1,NTRK3,OTX1,OTX2,RIMS1,SLC17A6,ST8SIA2,TBR1,TFAP2A,VAX1,WNT4, XRCC5
Cellular development	Differentiation of cells	3.5	ADAMTS20,ALOX12,ALOX5,ARL11,ASPN,ATOH1,AVPR1A,BHLHE22,BRINP1, BTG2,CAMP,CBLN1,CD1D,CD300LF,CD40LG,CDH23,CIITA,CNTN2,CR2,CSF3, CSRP3,CTNNA2,DCX,DISP3,DPYSL5,DSCAM,EBF3,ELAVL2,EPHB1,ERG,FABP2, FAS,FGF2,FGFR2,FLT1,FLT3,FOXD3,FSHB,GATA5,GDNF,GLP1R,GNAO1,GNAS, HAND1,HGF,HLA-DQA1,HNF1A,HTR2A,HTR2C,IFNA8,IFNK,IFNL3,IFNW1,IGF2, IGHG1,IGK,IKZF1,IKZF3,IL1RL1,IL20RA,IL23A,IL2RA,IRF6,KDF1,KRT84,LCK, LHX3,LHX6,LOR,LRRC7,MB,MDM2,MEOX1,mir-122,MYF6,MYH11,NCKAP1L,NFIX, NKX3-2,NLGN3,NMRK2,NR4A3,NRG1,NTRK3,OGN,OSTN,OTX1,OTX2,PARP1, PAX3,PAX5,PCSK2,PGR,PLG,POU2AF1,PRDM14,PRDM16,PRLR,PROK1,PROM1, PTPRC,RBM46,RIMS1,RIPK3,RIT2,S100B,SERPINB2,SFRP5,SHANK2,SHC4, SIGLEC10,SIT1,SLAMF1,SLIT1,SOX5,SPON1,ST8SIA2,TBL1X,TBR1,TBX1, TFAP2A,TFAP2B,TFAP4,THEMIS,THSD1,TRAT1,TRPM2,UBR2,USP28,VAX1, VEGFD,VPREB1,VWC2,WDR62,WNT16,WNT4,XRCC5
Behavior	Learning	3.0	ABI2,ADGRB3,AVPR1A,BRINP1,BTG2,CNTN2,CSF3,CUX2,CX3CR1,EPHA6,GJB6, GLP1R,GRIN2A,GRIN2B,HRH3,HTR2A,HTR2C,IGF2,KCNJ5,LRRC7,NLGN3,NRG1, NTRK3,PAH,PAK5,PCDH8,PEX5L,PPP1R1B,RPS6KA5,S100B,SHANK2,ST8SIA2, TBR1,TBX1,UNC13C
Behavior	Cognition	2.9	ABI2,ADGRB3,AVPR1A,BRINP1,BTG2,CNGA4,CNTN2,CSF3,CUX2,CX3CR1, EPHA6,GJB6,GLP1R,GNAS,GRIN2A,GRIN2B,HRH3,HTR2A,HTR2C,IGF2,KCNJ2, KCNJ5,LRRC7,NLGN3,NRG1,NTRK3,PAH,PAK5,PCDH8,PEX5L,PPP1R1B, RPS6KA5,S100B,SHANK2,ST8SIA2,TBR1,TBX1,UNC13C
Cellular development, cellular growth and proliferation, hematological system development and function, hematopoiesis, lymphoid tissue structure and development, tissue development	Development of leukocytes	2.8	BBC3,CD1D,CD40LG,CIITA,CR2,CSF3,CXCL11,EPHA3,EPHB1,FAS,FGF2,FLT1, FLT3,HLA-DQA1,HNF1A,IFNL3,IGF2,IKZF1,IL1RL1,IL23A,IL2RA,LCK,MSR1, MYH11,NCKAP1L,NPPA,PAX5,PGR,PLG,POU2AF1,PTPRC,RIPK3,SIT1,SLAMF1, SOX5,TFAP4,THEMIS,TRAT1,VPREB1,WNT4,XRCC5
Cellular development, nervous system development and function, tissue development	Differentiation of neurons	2.8	ATOH1,BHLHE22,BRINP1,BTG2,CBLN1,CDH23,CNTN2,DISP3,EBF3,ELAVL2, FGF2,FGFR2,GDNF,HGF,IGK,LHX3,NFIX,NTRK3,OTX2,PAX5,RIT2,ST8SIA2,TBR1, TBX1,TFAP2A,UBR2,VAX1,VEGFD,VWC2,WDR62
Nervous system development and function	Sensory system development	2.6	ABI2,ATOH1,ATOH7,CDHR1,CRYGB,CX3CR1,DSCAM,EBF3,EPHB1,FGF2,FGFR2,G[illegible]
Cellular development, cellular growth and proliferation, hematological system development and function	Cell proliferation of leukocyte cell lines	2.6	CSF3,FGF2,FGFR2,FLT1,FLT3,HIP1,IKZF1,IL22RA2,IL23A,IL23R,LCK, LOC102724788/PRODH,MBP,NRG1,NTRK3,PRDM16,PRLR,PTPRC
Cellular development	Differentiation of nervous system	2.5	ATOH1,BHLHE22,BRINP1,BTG2,CBLN1,CDH23,CNTN2,DISP3,EBF3,ELAVL2, FGF2,FGFR2,GDNF,HGF,IGK,KISS1R,LHX3,NFIX,NLGN3,NRG1,NTRK3,OTX2, PAX3,PAX5,RIT2,SOX5,ST8SIA2,TBR1,TBX1,TFAP2A,UBR2,VAX1,VEGFD,VWC2, WDR62,WNT4
Cellular development, hematological system development and function, hematopoiesis, lymphoid tissue structure and development	Differentiation of leukocytes	2.5	ALOX5,AVPR1A,CAMP,CD1D,CD40LG,CIITA,CR2,CSF3,FAS,FLT3,HGF,HLA-DQA1, HNF1A,IFNA8,IFNK,IFNL3,IFNW1,IGF2,IKZF1,IKZF3,IL23A,IL2RA,LCK,MYH11, NCKAP1L,PARP1,PAX5,POU2AF1,PRDM16,PTPRC,RIPK3,SFRP5,SIGLEC10,SIT1, SLAMF1,SOX5,TFAP4,THEMIS,TRAT1,TRPM2,VPREB1,WNT4,XRCC5
Cellular development, hematological system development and function, hematopoiesis, lymphoid tissue structure and development	Differentiation of mononuclear leukocytes	2.4	AVPR1A,CAMP,CD1D,CD40LG,CIITA,CR2,CSF3,FAS,FLT3,HGF,HLA-DQA1, HNF1A,IFNA8,IFNK,IFNL3,IFNW1,IGF2,IKZF1,IKZF3,IL23A,IL2RA,LCK,MYH11, NCKAP1L,PAX5,POU2AF1,PTPRC,RIPK3,SFRP5,SIGLEC10,SIT1,SLAMF1,SOX5, TFAP4,THEMIS,TRAT1,VPREB1,WNT4,XRCC5
Cellular development, cellular growth and proliferation, endocrine system development and function, organ development	Proliferation of pancreatic cells	2.4	FGF2,FOXD3,GAD2,GNAS,HGF,HNF1A,NR4A3,PGR,TPSD1

Categories[a]	Diseases or functions annotation	z-score[b]	Genes
Cellular growth and proliferation	Cell proliferation of hematopoietic cell lines	2.3	CSF3,FGF2,FGFR2,FLT1,FLT3,HIP1,IKZF1,IL22RA2,IL23A,IL23R,LCK, LOC102724788/PRODH,MBP,NRG1,NTRK3,PAX5,PRDM16,PRLR,PTPRC
Cellular growth and proliferation, lymphoid tissue structure and development	Proliferation of lymphatic system cells	2.3	ABI2,ACPP,BBC3,BTN1A1,CAMP,CD1D,CD40LG,CIITA,CLEC4D,CLEC4G,CR1L, CR2,CSF3,EPHB1,ERG,FAS,FCRL3,FGF2,FGL2,FLT3,GAD1,GAD2,HGF, HLA-DQA1,IFNL3,IGF2,IGHG1,IKZF1,IKZF3,IL1RL1,IL23A,IL23R,IL2RA,ITGAD, KLK13,KLRD1,LCK,MBP,MDM2,NCKAP1L,NPPA,PARP1,POU2AF1,PTPRC,RIPK3, RPS6KA5,SIGLEC1,SIGLEC10,SIT1,SLAMF1,THEMIS,TP53INP1,TYR,UBASH3A, VPREB1,VWF,XCL1,XRCC5
Cell death and survival, cellular compromise	Cytotoxicity of cytotoxic T cells	2.2	CD1D,CD40LG,IL23A,PTPRC,XCL1
Behavior	Conditioning	2.2	ABI2,CX3CR1,EPHA6,GAD2,GDNF,GJD2,GLP1R,GPR26,GRIK2,GRIN2A,NLGN3, NLRP6,PPP1R1B,RPS6KA5,SLC17A6,ST8SIA2,TBR1
Cellular movement	Mobility of cells	2.2	CGA,CSF3,FSHB,GAPDHS,NRG1
Behavior, nervous system development and function	Memory	2.2	AVPR1A,BRINP1,CSF3,CUX2,CX3CR1,GJB6,GLP1R,GRIN2A,GRIN2B,HRH3, HTR2A,IGF2,KCNJ5,LRRC7,NRG1,NTRK3,PAK5,PCDH8,S100B,SHANK2,TBX1
Cellular development, connective tissue development and function, tissue development	Differentiation of stromal cells	2.2	CD40LG,FGF2,GDNF,PARP1,WNT4
Embryonic development, organismal development, tissue development	Expansion of embryonic tissue	2.2	FGF2,HNF1A,MYF6,NRG1,REV3L
Cellular development, cellular growth and proliferation, endocrine system development and function	Proliferation of apud cells	2.2	FGF2,FOXD3,GAD2,GNAS,HGF,HNF1A,NR4A3,PGR
Auditory and vestibular system development and function, nervous system development and function	Hearing	2.0	ASIC2,CDH23,CLIC5,FOXI1,GJB6,GRID1,GRXCR2,KCNA1,MBP,OTOR,PAX3, TBL1X,TBX1,TECTB,TFAP2A
Gastrointestinal disease, organismal injury and abnormalities	Diarrhea	–2.0	ALOX12,ALOX5,CYP8B1,DEFA6,FGF2,GUCY2C,HTR3D,IL2RA,PTPRC,SSTR5
Cell death and survival	Apoptosis of granulosa cells	–2.2	BTG2,CGA,FAS,FGF2,HGF
Developmental disorder, immunological disease, organismal injury and abnormalities	Hypoplasia of lymph node	–2.2	CCRL2,CD40LG,SIT1,TRAT1,XRCC5
Gastrointestinal disease, inflammatory response, organismal injury and abnormalities	Inflammation of intestine	–2.3	ALOX12,ALOX5,CD40LG,CIITA,CR1L,CR2,CYP11B1,F10,FAS,FGFR2,GABRA6, GABRG3,GABRQ,GABRR2,GRIN2A,GRIN2B,GUCY2C,HGF,HLA-DQA1,IGF2, IL1RL1,IL22RA2,IL23A,IL23R,IL2RA,MUC2,NLRP6,PARP1,PGLYRP4,PLG,PTPRC, TP53INP1
Organismal injury and abnormalities, renal and urological disease	Failure of kidney	–2.4	CD1D,CR1L,CSF3,F10,FCGR1B,FLT1,GABRA6,GABRG3,GDNF,HGF,IGHG1,IL2RA, MAS1,NPPA,RRM2B,SSTR5,VKORC1,WFDC2,WNT4
Organismal injury and abnormalities, renal and urological disease	Glomerulosclerosis	–2.4	CD1D,CR1L,CX3CR1,GAD2,GATA5,GDNF,HGF,IGHG1,IL2RA,MAS1,RRM2B, WFDC2
Cellular compromise, ophthalmic disease, organismal injury and abnormalities, tissue morphology	Degeneration of photoreceptors	–2.4	CX3CR1,FGF2,GUCY2D,GUCY2F,IGF2,LRAT,OTX2,PROM1,RPGRIP1
Neurological disease, organismal injury and abnormalities	Seizure disorder	–2.6	BBC3,CACNG4,CNTN2,DCX,FLT1,GABRA6,GABRG3,GABRQ,GABRR2,GAD1, GAD2,GDNF,GFAP,GNAO1,GRIK2,GRIK3,GRIN2A,GRIN2B,HTR1E,HTR2A,HTR2C, HTR3D,KCNA1,KCNA4,KCNC1,KCNK1,NR4A3,NRG1,OTX1,OTX2,PGR,PPP1R1B, PTPRC,RIMS1,SCN2B,SLC13A1,SLITRK3
Gastrointestinal disease, inflammatory disease, inflammatory response, organismal injury and abnormalities	Colitis	–2.6	ALOX12,ALOX5,CD40LG,CIITA,CR1L,CR2,CYP11B1,F10,FAS,FGFR2,GABRA6, GABRG3,GABRQ,GABRR2,GRIN2A,GRIN2B,GUCY2C,HGF,HLA-DQA1,IL1RL1, IL22RA2,IL23A,IL23R,IL2RA,MUC2,NLRP6,PGLYRP4,PLG,PTPRC,TP53INP1
Infectious diseases	Bacterial infections	–2.7	CAMP,CCL8,CCRL2,CD1D,CLEC4D,CR2,CSF3,FCGR1B,FGFR2,GABRA6, GABRG3,GDF15,GRIN2A,GRIN2B,GUCY2C,IGK,IL23A,IL23R,IL2RA,ITGAD, LILRB5,MBL2,MSR1,NRG1,PARP1,PLG,RIPK3,RPS6KA5,SIGLEC1,SLAMF1, TLR10,TRPM2,VWF
Gastrointestinal disease, inflammatory disease	Gastroenteritis	–2.7	ALOX12,ALOX5,CD40LG,CIITA,CR1L,CR2,CYP11B1,F10,FAS,FGFR2,GABRA6, GABRG3,GABRQ,GABRR2,GRIN2A,GRIN2B,GUCY2C,HGF,HLA-DQA1,HTR3D, IL1RL1,IL22RA2,IL23A,IL23R,IL2RA,MUC2,NLRP6,PGLYRP4,PLG,PTPRC, TP53INP1,XCL1
Ophthalmic disease, organismal injury and abnormalities	Retinal degeneration	–2.7	ASIC2,CDH23,CDHR1,CIITA,CX3CR1,FGF2,GUCY2D,GUCY2F,HTR2A,IGF2,LRAT, NTRK3,OTX2,PROM1,RDH12,RDH8,RIMS1,RPGRIP1
Neurological disease, organismal injury and abnormalities	Seizures	–2.8	BBC3,CACNG4,CNTN2,GABRA6,GABRG3,GABRQ,GABRR2,GAD1,GAD2,GDNF, GFAP,GNAO1,GRIK2,GRIK3,GRIN2A,GRIN2B,HTR2C,KCNA1,KCNA4, KCNC1,KCNK1,NR4A3,NRG1,OTX1,PGR,PTPRC,RIMS1,SCN2B,SLC13A1, SLITRK3
Organismal injury and abnormalities, renal and urological disease	Urination disorder	–3.0	ASIC2,CD1D,CR1L,FAS,FCAR,FLT1,FXYD4,GATA5,GDF15,HGF,HNF1A,IKZF3, KCNK1,MAS1,MTRR,MYH11,NPPA,PGR,RRM2B,SLC13A1,VWF
Developmental disorder, organismal injury and abnormalities	Hypoplasia of lymphatic system	–3.7	CCRL2,CD40LG,IKZF1,LCK,MDM2,POU2AF1,PTPRC,RPS6KA5,RRM2B,SIT1, TBX1,TRAT1,VEGFD,XRCC5

Categories[a]	Diseases or functions annotation	z-score[b]	Genes
Inflammatory response, organismal injury and abnormalities	Inflammation of organ	–4.6	ABCB11,ACE2,ALOX12,ALOX5,BTN1A1,CAMP,CCL23,CCL8,CD1D,CD300LF, CD40LG,CIITA,CLEC4D,CLEC4G,CR1L,CR2,CSF3,CX3CR1,CXCL17,CYP11B1, CYP3A5,CYP3A7,F10,FAS,FCGR1B,FGFR2,FLT1,FLT3,GABRA6,GABRG3,GABRQ, GABRR2,GAD2,GATA5,GDNF,GFAP,GRIN2A,GRIN2B,GUCY2C,HGF,HLA-DQA1, HLA-DRB5,HMGCS2,HRH3,HTR2A,HTR2C,IFNL3,IGF2,IGHG1,IKZF3,IL1RL1, IL22RA2,IL23A,IL23R,IL2RA,KCNA1,KLK13,KLK6,KRT5,LCK,LOR,MBP,MDM2, mir-122,MUC2,NLRP6,NRG1,PARP1,PGLYRP4,PGR,PLG,PNLIP,PROM1,PTPRC, RDH8,RIPK3,RPS6KA5,RXRG,SERPINB3,SIGLEC10,SIT1,SOX5,TLR10,TP53INP1, TYR,UBASH3A,VEGFD,VWF,WNT4,XCL1

Information on the experimental condition is provided in the legends to S2 Fig.

[a] Analyese were conducted for 1265 genes that changed at $p < 0.0073$. Blue area highlights annotations that also yielded the z-score of >2 or < –2 for 2234 genes whose expression changed at $p < 0.0126$.

[b] Annotations with the z-score of >2 (indicative of activation) or < –2 (indicative of inhibition) are listed.
