# Supplementary material for: Ionizing radiation response of primary normal human lens epithelial cells
Source: PLoS One. 2017 Jul 26;12(7):e0181530. doi: 10.1371/journal.pone.0181530 (PMC5528879; doi:10.1371/journal.pone.0181530)
Supplement: S11 Table — (PDF) [file pone.0181530.s014.pdf]

S11 Table. Upstream regulators suggested in HLEC1 at 3 h after 4 Gy vs after 0 Gy.

Upstream Regulator[a]	Molecule Type	z-score[b]	Target genes in dataset
HNF1A	Transcription regulator	3.5	ACE2,ANKS4B,CLIC5,FUT5,FXYD3,G6PC2,GLP1R,HAL,HNF1A,HNF4G,PAH,PKHD1,PLA1A,PPP1R1A,PRLR,SLC17A2,TRPM2
NFkB (complex)	Complex	3.1	BBC3,CAMP,CCL8,CIITA,CSF3,CXCL11,CYP3A5,FAS,GAD1,GAD2,GDF15,GNAS,HIP1,IL23A,IL2RA,MBL2,MDM2,RAB3C,SERPINB2
TP53	Transcription regulator	2.6	ADGRB3,BBC3,BTG2,EGFL6,FAM212B,FAS,FDXR,FGF2,FXYD3,GDF15,HLA-DQA1,IGF2,IL2RA,LOC102724788/PRODH,MDM2,PADI2,PARD6G,PCNA,PHLDA3,PROM1,PSRC1,RPRM,RRM2B,SESN1,SESN2,TBL1X,TP53INP1,WNT2,XRCC5
IL21	Cytokine	2.6	CD1D,IGHG1,IL23R,IL2RA,KLRD1,PCNA,PTPRC
SLC29A1	Transporter	2.4	FAS,FDXR,MDM2,RRM2B,SESN1,TP53INP1
APP	Other	2.4	ANKS4B,ARL11,DCX,FGF2,GDNF,GFAP,GNAO1,IGF2,MBP,MSR1,OGN,PAX3,PAX5,PGK2,PRLR,PTPRC,S100B
CNR1	G-protein coupled receptor	2.3	APCDD1,FGF2,GAD1,GDNF,PAX3,PAX5,PCNA,RXRG,S100B
DTNBP1	Other	2.2	GAD2,GRIN2A,GRIN2B,LHX6,NPPA
Estrogen receptor	Group	2.2	CDH10,FGFR2,KISS1R,PCDH8,PGR,PRLR
ID3	Transcription regulator	2.2	CD40LG,FSHB,IKZF3,IRF6,NR4A3,SLAMF1,SOX5
ID2	Transcription regulator	2.2	CD40LG,FSHB,IKZF3,IRF6,NR4A3,SLAMF1,SOX5
PTF1A	Transcription regulator	2.2	ATOH1,CRABP1,GAD1,GAD2,GRIK2,GRIK3,MPPED2,TFAP2B
LHX1	Transcription regulator	2.1	C7orf57,GAD1,KDM5B,KLK6,OTX2,PAH,TMEM229A,TMIGD1
EBF2	Transcription regulator	2.0	LGI2,MEOX1,RBM46,SHISA6
PRDM8	Transcription regulator	–2.0	BHLHE22,EBF3,EPHA6,GJD2
ANXA2	Other	–2.0	FAS,MDM2,SESN1,SESN2
NR1H3	Ligand-dependent nuclear receptor	–2.0	CSF3,DIO2,PRDM16,TBX1
NR1H2	Ligand-dependent nuclear receptor	–2.0	CSF3,DIO2,PRDM16,TBX1
NOS2	Enzyme	–2.1	CLEC4D,GDNF,IGHG1,LCK,LOR,MB,NPPA,PCNA,RPTN
COMMD3-BMI1	Transcription regulator	–2.2	HOXA13,HOXB6,HOXC9,POU2AF1,VPREB1
COL18A1	Other	–2.4	EPHB1,F10,FGF2,GFAP,HGF,VWF

Information on the experimental condition is provided in the legends to S2 Fig.

[a] Analyses were conducted for 1265 genes that changed at $p < 0.0073$. Blue area highlights upstream regulators that also yielded the z-score of >2 or < –2 for 2234 genes whose expression changed at $p < 0.0126$.

[b] Upstream regulators with the z-score of >2 (indicative of activation) or < –2 (indicative of inhibition) are listed.
